# MD2 deficiency prevents high‐fat diet‐induced AMPK suppression and lipid accumulation through regulating TBK1 in non‐alcoholic fatty liver disease

**DOI:** 10.1002/ctm2.777

**Published:** 2022-03-28

**Authors:** Wu Luo, Lin Ye, Xue‐ting Hu, Mei‐hong Wang, Min‐xiu Wang, Lei‐ming Jin, Zhong‐xiang Xiao, Jian‐chang Qian, Yi Wang, Wei Zuo, Li‐jiang Huang, Guang Liang

**Affiliations:** ^1^ School of Pharmaceutical Sciences Hangzhou Medical College Hangzhou China; ^2^ Chemical Biology Research Center School of Pharmaceutical Sciences Wenzhou Medical University Wenzhou China; ^3^ Medical Research Center, The First Affiliated Hospital Wenzhou Medical University Wenzhou China; ^4^ Affiliated Yueqing Hospital Wenzhou Medical University Yueqing China; ^5^ Affiliated Xiangshan Hospital Wenzhou Medial University (Xiangshan First People's Hospital Medical and Health Group) Xiangshan China

**Keywords:** AMPK/SREBP1, lipid accumulation, MD2, NAFLD, TBK1

## Abstract

**Background:**

Non‐alcoholic fatty liver disease (NAFLD) is the most predominant form of liver diseases worldwide. Recent evidence shows that myeloid differentiation factor 2 (MD2), a protein in innate immunity and inflammation, regulates liver injury in models of NAFLD. Here, we investigated a new mechanism by which MD2 participates in the pathogenesis of experimental NAFLD.

**Methods:**

Wild‐type, *Md2* ^−/−^ and bone marrow reconstitution mice fed with high‐fat diet (HFD) were used to identify the role of hepatocyte MD2 in NAFLD. Transcriptomic RNA‐seq and pathway enrich analysis were performed to explore the potential mechanisms of MD2. In vitro, primary hepatocytes and macrophages were cultured for mechanistic studies.

**Results:**

Transcriptome analysis and bone marrow reconstitution studies showed that hepatocyte MD2 may participate in regulating lipid metabolism in models with NAFLD. We then discovered that *Md2* deficiency in mice prevents HFD‐mediated suppression of AMP‐activated protein kinase (AMPK). This preservation of AMPK in *Md2*‐deficient mice was associated with normalized sterol regulatory element binding protein 1 (SREBP1) transcriptional program and a lack of lipid accumulation in both hepatocytes and liver. We then showed that hepatocyte MD2 links HFD to AMPK/SREBP1 through TANK binding kinase 1 (TBK1). In addition, MD2‐increased inflammatory factor from macrophages induces hepatic TBK1 activation and AMPK suppression.

**Conclusion:**

Hepatocyte MD2 plays a pathogenic role in NAFLD through TBK1‐AMPK/SREBP1 and lipid metabolism pathway. These studies provide new insight into a non‐inflammatory function of MD2 and evidence for the important role of MD2 in NALFD.

## Graphical abstract

Hepatocyte MD2 plays a pathogenic role in NAFLD through TBK1‐AMPK/SREBP1 and lipid metabolism pathway.
PA‐MD2 interaction activates TBK1, which directly inhibit AMPK to increase the downstream SREBP1 activity and lead to hepatic lipid accumulation in hepatocytes.
(3) MD2‐mediated inflammatory factors in macrophages also contribute to hepatocyte lipid accumulation and injury via TBK1‐AMPK pathway.

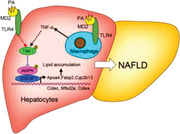

## 1 INTRODUCTION

Non‐alcoholic fatty liver disease (NAFLD) is the most predominant form of liver diseases worldwide.^1^, ^2^, ^3^ A hallmark of NAFLD is ectopic lipid accumulation in livers, which early on, may occur with minimal hepatocellular injury. Although diet/exercise may provide some benefit in NAFLD,^4^ there is currently no drug therapy. Multiple sources contribute to the intrahepatic triglyceride accumulation in patients with NAFLD, including hepatic uptake from plasma and de novo lipogenesis.^5^ Increasing evidence also indicates that inflammation contributes greatly to the pathogenesis of NAFLD.^6^, ^7^

As a pattern recognition receptor in innate immunity, toll‐like receptor 4 (TLR4), together with its assistant protein myeloid differentiation factor 2 (MD2), can be activated by microbial endotoxin or endogenous free fatty acids to produce pro‐inflammatory cytokines. Once MD2/TLR4 is activated by ligands, intracellular adapter proteins including TIR‐domain‐containing adapter‐inducing interferon‐β (TRIF) and myeloid differentiation primary response factor‐88 (MyD88) are recruited to TLR4. Signaling through the TRIF pathway is well‐characterized and involves cascading activation of TANK binding protein 1 (TBK1) and interferon regulatory factor‐3.^8^ Recent studies have identified an important function of MD2/TLR4 in the progression of NAFLD.^9^ Our group has previously shown that *Md2* deficiency or pharmacological MD2 inhibition prevents lipid accumulation, pro‐fibrotic change and pro‐inflammatory molecule expression in the livers of high‐fat diet (HFD)‐fed mice.^10^ Mechanistically, similar readouts, such as fibrosis and inflammatory factor induction, can be recapitulated in culture systems exposed to palmitate (PA).^11^ In addition, we recently discovered that PA directly binds to MD2 to induce cellular inflammation.^12^ Similarly, other groups have shown that diets deficient in methionine choline (MCD) increases liver triglycerides accumulation and production of inflammatory factors in mice.^13^ Such alterations are not seen in *Md2‐/‐* or *Tlr4‐/‐* mice.^13^ These studies show that MD2 may play a critical pathogenic role in NAFLD.

Based on the robust expression of TLR4 on many cell types in the liver, including Kupffer cells (KCs), hepatocytes and stellate cells,^14^ and the typical pro‐inflammatory role of TLR4/MD2 pathway,^15^, ^16^ it is generally believed that MD2 mediates inflammatory responses in NAFLD. Although this may be correct, contribution of other mechanisms has not been ruled out. For example, pro‐oxidant effects of some lipid species, such as PA, also greatly contribute to the hepatic toxicity.^17^ Furthermore, transcriptome studies in HFD fed mice shows pathways associated with lipid metabolism to be predominant, along with inflammatory responses.^18^ Therefore, further studies are needed to understand how *Md2* deficiency affords protection in NAFLD. These studies are critical for the development of effective therapy for NAFLD. Here, we identified lipid metabolism pathways regulated by MD2 through transcriptome analysis using Messenger Ribose Nucleic Acid sequencing (mRNA sequencing) (RNA‐seq). We show that *Md2* deficiency prevents HFD‐mediated suppression of AMP‐activated protein kinase (AMPK), reducing sterol regulatory element binding protein 1 (SREBP1) transcriptional program and lipid accumulation in the liver.

## 2 MATERIALS AND METHODS

### 2.1 Reagents

Antibodies against MD2 (cat# sc‐80183) and SREBF1 (cat# sc‐365513) were obtained from Santa Cruz Biotechnology (Texas, United States). Antibodies for AMPK (cat# 5831), phosphorylated (p‐) AMPK (cat# 4188), TBK1 (cat# 5483) and p‐TBK1 (cat# 51872) were obtained from Cell Signaling Technology (Massachusetts, United States). Antibodies for glyceraldehyde‐3‐phosphate dehydrogenase (GAPDH) (cat# AB‐P‐R001) was from Hangzhou Goodhere Biotechnology (Hangzhou, Zhejiang, China). Antibodies for albumin (cat# ab106582) and macrophage marker F4/80 (cat# ab6640) were purchased from Abcam (Cambridge, United Kingdom). Alexa‐647 and ‐488 conjugated secondary antibodies and Horseradish peroxidase‐conjugated secondary antibodies were purchased from Abcam and Santa Cruz Biotechnology, respectively. Diaminobenzidine (DAB) and Pierce ECL Western Blotting Substrate were purchased from Thermo Fisher (Massachusetts, USA).

Assay kits for aspartate aminotransferase (AST), alanine aminotransferase (ALT), low‐density lipoprotein‐cholesterol (LDL‐C), triglyceride (TG), total cholesterol (TCH) and high‐density lipoprotein‐cholesterol (HDL‐C) were obtained from Nanjing Jiancheng BioInstitute (Nanjing, China). Hematoxylin and eosin (H&E) staining kit (cat# G1120) and oil‐red O staining solution (cat# G1261) were obtained from Solarbio Life Sciences (Beijing, China). Bovine serum albumin (BSA; cat# A1933) and sodium PA (cat# P9767‐5G) were purchased from Sigma (Missouri, United States). A working solution of 5 mM PA was prepared by heating PA to 80°C and adding it to 5% BSA maintained at 55°C. 5‐aminoimidazole‐4‐carboxamide riboside (AICAR; cat# HY‐13417), a direct AMPK activator, was obtained from MedChemExpress (New Jersey, United States). A selective TBK1 inhibitor GSK8612 (Cat# S8872) was purchased from Selleck (Shanghai, China). Levels of tumour necrosis factor‐α (TNF‐α) in cell medium and serum were measured with mouse TNF‐α ELISA (cat# 85‐88‐7324‐76; eBioscience, CA, United States). Collagenase type 2 (cat# LS004176) were obtained from Worthington Biochemical Corp (New Jersey, United States).

### 2.2 Cell culture models

Mouse primary peritoneal macrophages (MPMs) were isolated from wildtype MD2^−/−^ mice and C57BL/6 mice, respectively, as described in our previous paper.^11^ This study utilized primary hepatocytes and a hepatocyte line. Primary hepatocytes, hepatic stellate cells (HSCs), non‐parenchymal cells (NPCs), KCs and hepatic endothelial cells (ECs) were isolated from mice livers by a combination of magnetic activated cell sorting and collagenase‐based density gradient centrifugation, as described in previous study.^19^ Mouse hepatocyte cell line AML12 is just the name of a hepatic cell line (AML‐12) (cat# SCSP‐550) was purchased from Shanghai Institute of Biochemistry and Cell Biology (Shanghai, China) and maintained in Dulbecco's Modified Eagle Medium (DMEM)/F12K (cat# SH30023.01; Gibco) with 10% fetal bovine serum (FBS), 40 ng/ml dexamethasone, 1x insulin‐transferrin‐selenium, 100 U/ml penicillin and 100 mg/ml streptomycin.

### 2.3 Mouse model of NAFLD

Mouse study protocol was approved by the Animal Policy and Welfare Committee of Wenzhou Medical University (approval number: wydw2019‐0018). Sixteen male MD2^−/−^ mice on a C57BL/6 background (B6.129P2‐Ly96 < tm1Kmiy > ; myeloid differentiation protein 2 gene knockout (MD2KO); confirmed as shown in Figure S1) were purchased from Riken BioResource Center of Japan (Tsukuba, Ibaraki, Japan). Forty male C57BL/6 mice (substrain background B6.129P2) at 8 weeks of age were obtained from GemPharmatech (Nanjing, China). Mice were housed with a 12:12‐h light–dark cycle at 22°C. A standard low‐fat rodent diet (LFD, containing 70 kcal.% carbohydrate, 10 kcal.% fat, and 20 kcal.% protein; Cat. #MD12031) and an HFD (including 20 kcal.% carbohydrate, 60 kcal.% fat, and 20 kcal.% protein; Cat. #MD12033) were purchased from Mediscience Diets Co. LTD (Yangzhou, China). One week after acclimatization, 12 male MD2^−/−^ mice were divided randomly into control (MD2KO‐LFD) and MD2KO‐HFD groups (*n* = 6 per group), and 12 age‐matched male C57BL/6 mice were divided randomly into wild type (WT)‐LFD and WT‐HFD groups (*n* = 6 per group). WT‐LFD and MD2KO‐LFD groups feed with LFD for 16 weeks, while WT‐HFD and MD2KO‐HFD groups received HFD for 16 weeks. Body weight (BW) was recorded weekly during the experimental period. Sixteen weeks after treatment, the mice were sacrificed under anaesthesia. The liver tissues and blood samples were collected. Total serum triglyceride, LDL‐C, cholesterol and HDL‐C were measured using commercial kits. For liver triglyceride and cholesterol levels, tissues were homogenized in ethanol‐acetone, and the suspension was used to measure cholesterol and triglycerides.

### 2.4 Bone marrow transplantation

Six‐ to eight‐week‐old male recipient mice (WT and MD2KO) were put in filter‐top cages and given sterile water containing polymyxin B sulphate (1000 U/L) and neomycin (1.1 mg/L) for 1 week before transplantation. The recipient mice were performed to total body irradiation (6 Gy) 12 h before transplantation. To harvest the bone marrow cells, femurs and tibias were obtained from 6–8‐week‐old male WT or MD2KO donor mice. Bone marrow was flushed out with a 24‐gauge syringe, and a single cell suspension was obtained by filtering the suspension with a 100‐μm nylon filter after red blood cell lysis. Bone marrow cells (2 × 10^6^) were transferred intravenously into the irradiated recipient mice. The mice were allowed to rest for 4 weeks and then fed with HFD for 16 weeks (*n* = 6 per group).

### 2.5 Human liver samples

Procedures involving human clinical samples followed the principles in the Declaration of Helsinki. These procedures were approved by the Human Ethics Committee of the First Affiliated Hospital of Wenzhou Medical University (approval number: 2019–032). Hepatic steatotic specimens (*n* = 6) were collected from non‐alcoholic steatohepatitis (NASH) patients who had undergone liver biopsy in the First Affiliated Hospital of Wenzhou Medical University. The nonsteatotic liver samples (*n* = 6) were obtained from patients diagnosed with liver haemangioma and hepatic cyst and accepted liver surgeries in the same hospital. The clinical information and histological features of non‐NASH individuals and NASH patients are shown in Table S1.

### 2.6 Cell and tissue staining

Portions of harvested liver tissues were fixed in paraformaldehyde for paraffin embedding and in optimal cutting temperature media for staining requiring frozen tissues. Paraffin tissue sections at 5‐μm thickness were subjected to routine H&E staining anaylsis. The quantitative severity of NAFLD from H&E staining images was determined using NAFLD activity score (NAS) system, which has been widely recognized and used for experimental NAFLD/NASH studies.^20^

Sections were deparaffinized, rehydrated and blocked with 3% H_2_O_2_ for 30 min and in 1% BSA for 30 min for immunohistochemical staining. Primary antibody against MD2 (1:300) was applied overnight at 4°C. After applied with peroxidase‐conjugated secondary antibody for 1 h, the immunoreactivity was detected with DAB. As a control, sections were counterstained with hematoxylin. A Nikon microscope (Nikon, Japan) was used to collect images.

For oil‐red O staining, we prepared 10‐μm thick frozen sections, which were fixed in formalin solution (10%) for 10 min and dehydrated in propylene glycol at room temperature for 10 min. Then, sections were stained with oil‐red O solution for 10 min. A 60% propylene glycol solution was added for 1 min. Nuclei were stained in hematoxylin. Oil red O staining of fixed cells was carried out using the same protocol.

Immunofluorescence staining of liver tissues was performed on 5‐μm thick frozen sections. Briefly, sections were fixed in formalin for 10 min, blocked with BSA at 1% for 1 h and then incubated with primary antibodies overnight. Fluorophore‐conjugated secondary antibodies were then incubated at room temperature for 1 h. Sections were also counterstained with 4′,6‐diamidino‐2‐phenylindole (DAPI) and imaged by an epi‐fluorescence microscope (Nikon).

### 2.7 Western blot and co‐immunoprecipitation

Total proteins from tissues or primary hepatocyte were prepared using Radio Immunoprecipitation Assay (RIPA) buffer (cat# P0013B; Beyotime Biological Technology, Shanghai, China). Total proteins in samples were measured with bicinchoninic acid (BCA) (cat# 23225; Thermo Scientific). Approximately 40–80‐μg protein samples were subjected to 10% sodium dodecyl sulfate polyacrylamide gel electrophoresis (SDS‐PAGE) and then transferred to polyvinylidene fluoride membranes. The transferred membranes were blocked in a routine Tris‐buffered saline for 1.5 h at room temperature before applying primary antibodies. Protein bands were visualized by secondary antibodies and enhanced chemiluminescence reagent (Thermo Scientific). Band densities normalized to loading controls were quantified using Image J software.

In co‐immunoprecipitation assays, cellular total protein was extracted using cell lysis buffer for immunoprecipitation (IP) (cat# P0013; Beyotime) containing protease inhibitor cocktail. Extracts were incubated with AMPK antibody at 4°C overnight. Samples were added to A/G‐Sepharose beads at 4°C for 2 h. The beads were washed with Tris‐HCl buffered saline (TBS) for five times, and the proteins were then eluted with SDS loading buffer at the temperature of 95°C. Immunoblotting was performed to detect proteins.

### 2.8 Real‐time quantitative polymerase chain reaction (qPCR) assay

Messenger Ribose Nucleic Acid (mRNA) levels were examined by routine real‐time qPCR assay. Total Ribose Nucleic Acid (RNA) from cells and tissues were extracted, respectively, using TRIzol kit (Thermo Fisher). One‐microgram RNA was used for reverse transcription using the PrimeScript RT reagent with gDNA Eraser (Takara; cat# RR047A). qPCR was performed with iQ SYBR Green Supermix (Bio‐Rad; cat# 1708882) and QuantStudio 3 Real‐Time PCR System (Bio‐Rad). Relative mRNA expression was normalized with *Actb*. Primer sequences are shown in Table S2.

### 2.9 Transcriptome sequencing and analysis

A genome‐wide analysis was performed using fresh liver tissues from mice by Lianchuan Bio Technologies (Hangzhou, Zhejiang). Paired‐end sequencing was performed using the Illumina Novaseq 6000 (California, United States). Bioinformatic analysis on RNA‐sequencing data was also performed by Lianchuan Bio Technologies (Hangzhou, Zhejiang). The differentially expressed mRNAs with fold change >2 or fold change <.5 and *p*‐value <.05 were chosen by DESeq2 at http://www.bioconductor.org/packages/release/bioc/html/DESeq2.html and R package edgeR at https://bioconductor.org/packages/release/bioc/html/edgeR.html. GSEA was performed by GSEA4.0 soft (http://software.broadinstitute.org/gsea/downloads.jsp). Transcription factor prediction and enriched pathway analysis were performed by modEnrichr soft (https://amp.pharm.mssm.edu/modEnrichr/).

**FIGURE 1:**
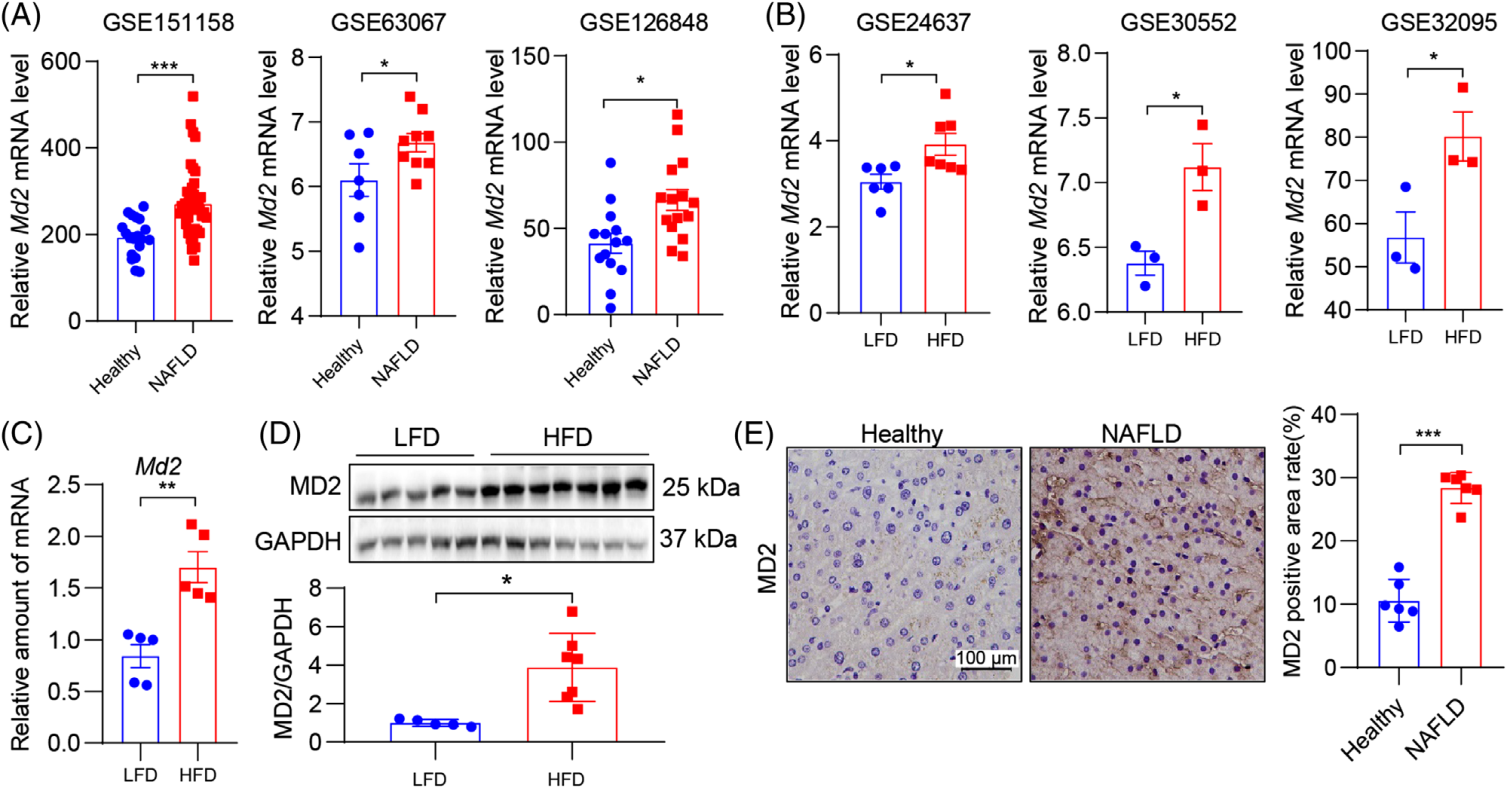
MD2 levels are elevated in human non‐alcoholic fatty liver disease (NAFLD) and experimental models. (A) Dot plots showing relative MD2 mRNA levels in liver tissues of control (healthy) subjects and patients with NAFLD. Data retrieved from GSE151158, GSE63067 and GSE126848. Comparisons were analysed by *t*‐test. (B) Dot plots showing relative MD2 mRNA levels in liver tissues of mice fed a control/low‐fat diet (LFD) and high‐fat diet (HFD). Data retrieved from GSE24637, GSE30552 and GSE32095. Comparisons were analysed by *t*‐test. (C) mRNA levels of MD2 in liver tissues of mice fed an LFD or HFD for 16 weeks. Data normalized to *Actb* (*n* = 6; mean ± standard error of mean (SEM)). (D) Protein levels of MD2 were detected in mouse liver tissue lysates. GAPDH was used as loading control. Densitometric quantification is shown below the blots (*n* = 5–7; mean ± SEM). (E) Representative immunostaining of human liver tissues for MD2 (brown). Tissues were obtained from control subjects and patients with NAFLD. Counterstaining with hematoxylin (blue) was performed (scale bars = 100 μm). Densitometric quantification is shown on right (*n* = 6; mean ± SEM). ns, not significant. *p < .05; **p < .01; ***p < .001

For some studies in Figure 1, we utilized publicly available transcriptome data. Data from studies related to NAFLD mice were retrieved from the Gene Expression Omnibus database in www.ncbi.nlm.nih.gov/geo/. Search terms included NAFLD (All Fields) AND Homo sapiens/Mus musculus (porgn). Six series of datasets, GSE151158,^21^ GSE63067,^22^ GSE126848,^23^ GSE24637,^24^ GSE30552^25^ and GSE32095^26^ were selected. MD2 levels were determined using data analysis tools in the expression profiling by array data or gene ID convert tools in expression profiling by high throughput sequencing data.

### 2.10 Chromatin immunoprecipitation

Primary hepatocytes from MD2KO and WT mice were expanded and used for chromatin IP using the SimpleChIP enzymatic chromatin IP kit (cat# 9003; Cell Signaling Technology). Samples were incubated with SREBP1 antibody at 4°C overnight, and then the Protein A/G agarose was added. As a negative control, IgG antibody was used in parallel. qPCR was performed to detect *Apoa4* and *Cidea* promoter‐specific regions. Primers are listed in Table S2.

### 2.11 Oxygen consumption rate analysis

A Seahorse XF‐24 Extracellular Flux Analyzer (Seahorse Bioscience, North Billerica, MA) was used to measure the intact cellular oxygen consumption rate in PA‐treated primary hepatocytes. Briefly, 500‐μl single‐cell suspensions of 10^5^ primary hepatocytes cells were plated in XF24 cell culture microplates (four replicates of each group of cells). Cells were incubated in base assay medium supplemented with 10 mM glucose, 1 mM pyruvate and 2 mM glutamine for 1 h, before measured by the XF Cell Mito Stress Kit (Seahorse Bioscience).

### 2.12 Statistical analysis

All data are presented as mean ± SEM, and the statistical significance was calculated using GraphPad Prism 8.0 software (San Diego, CA). The Student's *t*‐test was used to determine the *p* values for normally distributed data. One‐way Analysis of Variance (ANOVA) and two‐way ANOVA with Bonferroni post‐test were used to analyse multiple groups with only one variable tested and more than two groups with multiple variables tested, respectively. *p‐*Value <.05 was considered significant.

## 3 RESULTS

### 3.1 *Md2* is elevated in liver tissues of subjects with NAFLD and in experimental models of the disease

We first examined the levels of *Md2* in human NAFLD and in experimental models. We found three human studies and three studies in experimental models that examined liver tissues with transcriptome analyses. In human studies, liver tissues of subjects with NAFLD activity (GSE151158,^21^ GSE63067^22^ and GSE126848^23^) showed elevated levels of *Md2* mRNA compared to health/NALFD‐activity‐negative samples (Figure 1A). Similarly, experimental studies of TALLYHO/JngJ (GSE24637^24^), or HFD‐fed 129 Sv (GSE30552^25^) or C57BL/6 (GSE32095^26^) mice showed elevated *Md2* gene expression in liver compared to respective controls (Figure 1B). Consistent with these studies, HFD feeding of C57BL/6 mice for 16 weeks in the present study showed increased *Md2* mRNA (Figure 1C) and MD2 protein (Figure 1D). We also confirmed increased MD2 protein levels in human subjects with NAFLD (Figure 1E). Collectively, these studies show that expression of MD2 is increased in NAFLD and warrant elucidation of the mechanisms by which MD2 may mediate NAFLD pathogenesis.

### 3.2 Hepatocyte MD2 mediates HFD‐induced lipid accumulation and liver injury

**FIGURE 2:**
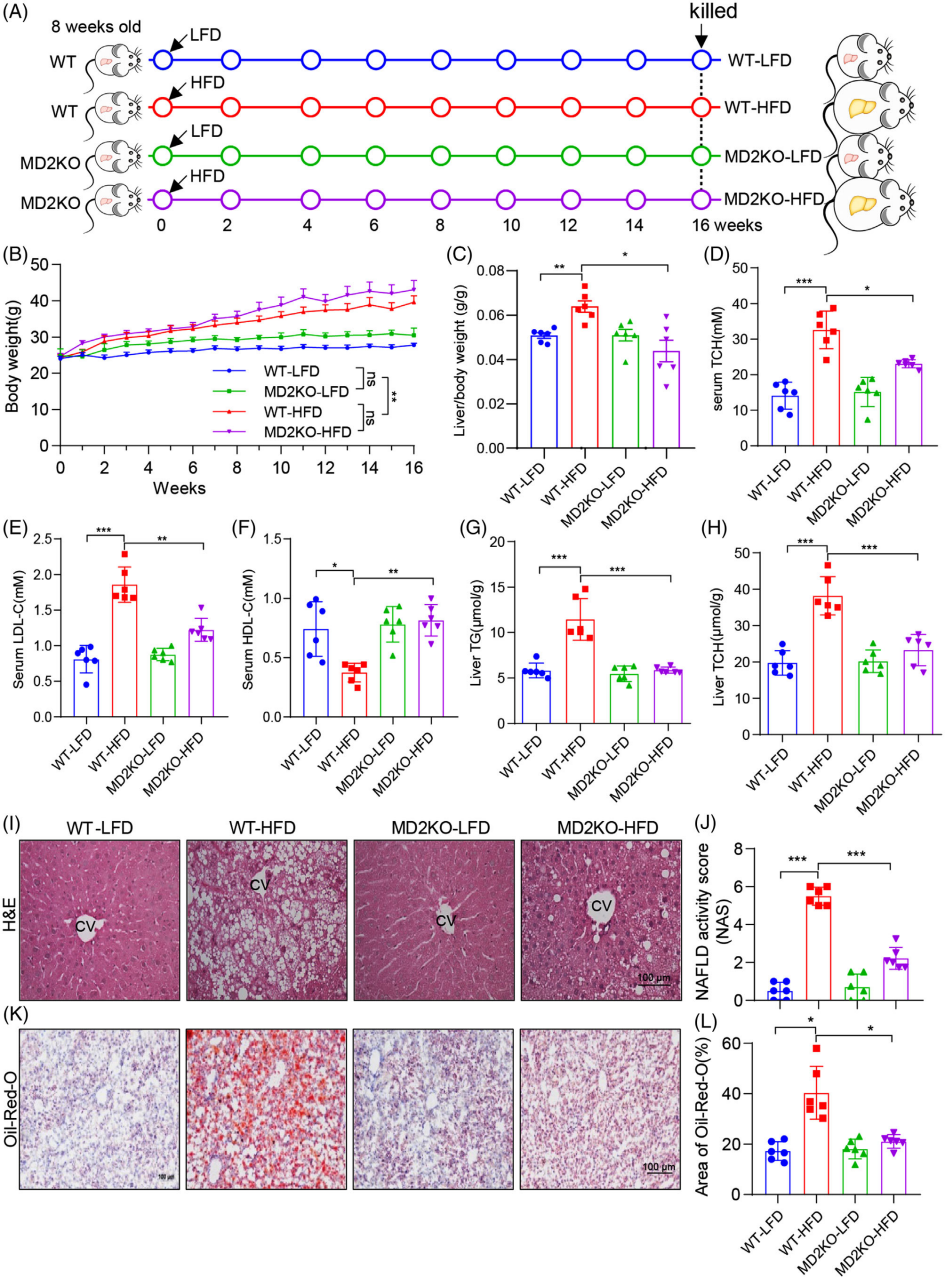
MD2 knockout mice display reduced lipid accumulation in liver tissues when fed a high‐fat diet. (A) The schematic diagram showing the grouping and steps of animal experiments. WT and MD2KO mice were fed with low‐fat rodent diet (LFD) or high‐fat diet (HFD) for 16 weeks. (B) Body weight of mice measured weekly (*n* = 6; mean ± SEM; ns, not significant; **p < .01]. (C) Ratios of liver weight relative to body weight (LW/BW) at the time of harvest. (D–F) Serum levels of total cholesterol (TCH) (D), low‐density lipoprotein‐cholesterol (LDL‐C) (E) and high‐density lipoprotein‐cholesterol (HDL‐C) (F) in mice. (G and H) Levels of triglyceride (TG) (G) and TCH (H) in liver tissues of mice. (I and J) Assessment of liver injury as determined by hematoxylin and eosin (H&E) staining (I) and non‐alcoholic fatty liver disease (NAFLD) activity score analysis (J) (scale bars = 100 μm). (K and L) Frozen liver tissues were stained with Oil red O to assess lipid accumulation. Representative images are shown in panel K (scale bars = 100 μm). Quantification is shown in panel (L). In panel C–L, *n* = 6 per group; data are shown in mean ± SEM. *p < .05; **p < .01; ***p < .001

To understand the mechanistic role of MD2 in NAFLD, we fed WT and *Md2^−/−^* (MD2KO) mice an HFD for 16 weeks and compared them to mice fed a control LFD (Figure 2A). HFD feeding increased BWs of mice as expected (Figure 2B). There was no difference between WT and MD2KO, when compared within the specific diet group. The ratios of liver weight relative to BW (LW/BW) showed that MD2 knockout normalized the increased weight of liver tissues in HFD‐fed mice (Figure 2C). Analysis of serum and liver lipid profile showed that MD2KO mice are protected against HFD‐mediate dyslipidemia. HFD‐fed mice exhibited increased levels of serum TCH, serum LDL‐C, liver TG and liver TCH, and serum HDL‐C level was decreased by HFD feeding (Figure 2D–H). These changes of lipid levels induced by HFD were significantly reversed by MD2 gene knockout (Figure 2D–H). Liver tissues of WT mice fed an HFD showed features of NAFLD including, steatosis and injury severity, as evident in H&E sections and NAS analysis (Figure 2I,J). This injury level was not seen in MD2KO mice fed an HFD. We then assessed the liver function by measuring serum AST and ALT levels. Serum Levels of ALT and AST were significantly increased in WT mice on HFD, but not in MD2KO mice suggesting that MD2 deficiency prevented liver function injury (Figure S2). Similarly, staining frozen liver tissues with oil red O demonstrated that the hepatic lipid accumulation was remarkably lower in HFD‐fed MD2KO, compared to WT mice on the same diet (Figure 2K,L). These studies confirm our previous reports of protection afforded by *Md2* deficiency against HFD‐induced liver injury.^10^

**FIGURE 3:**
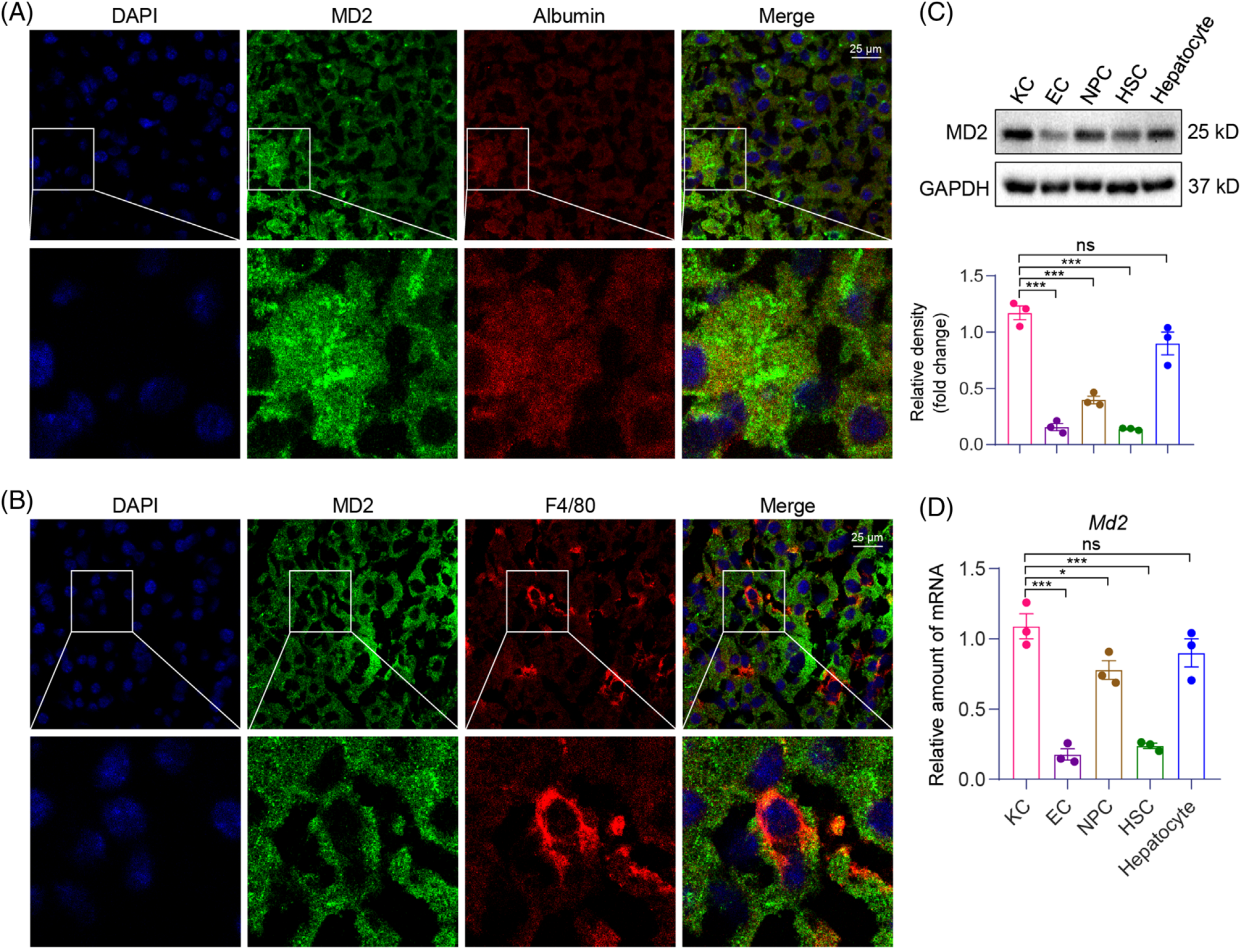
MD2 protein localizes to hepatocytes and hepatic macrophages in liver tissues. (A) Representative immunofluorescence staining images of normal mouse liver tissues showing immunoreactivity to MD2 (green) and hepatocyte‐marker albumin (red). Tissues were counterstained with DAPI (blue). Arrows indicating co‐localization (scale bars = 25 μm). (B) Representative staining images showing immunoreactivity to MD2 (green) and macrophage marker F4/80 used to detect hepatic macrophages (red). Tissues were counterstained with DAPI (blue). Arrows indicating co‐localization (scale bars = 25 μm). (C and D) Primary Kupffer cells (KC), endothelial cells (ECs), non‐parenchymal cells (NPC), hepatic stellate cells (HSCs) and hepatocytes were isolated from mouse livers. The protein (C) and mRNA (D) levels of MD2 in these cells were examined by western blot and qPCR assay, respectively (*n* = 3; mean ± SEM; ns, not significant; *p < .05, **p < .01 and ***p < .001)

We stained the liver tissues of mice for MD2 to determine the main cellular source of MD2. We found that both albumin‐positive hepatocytes and F4/80‐positive myeloid cells robustly express MD2 (Figure 3A,B). This is the first time to show that hepatocytes express abundant MD2 protein. We further validate the MD2 expression distribution using primary HSCs, KCs, hepatocytes, ECs and NPCs isolated from mouse livers. Both western blot and qPCR assays showed that MD2 is mainly expressed in myeloid cells and hepatocytes (Figure 3C,D).

**FIGURE 4:**
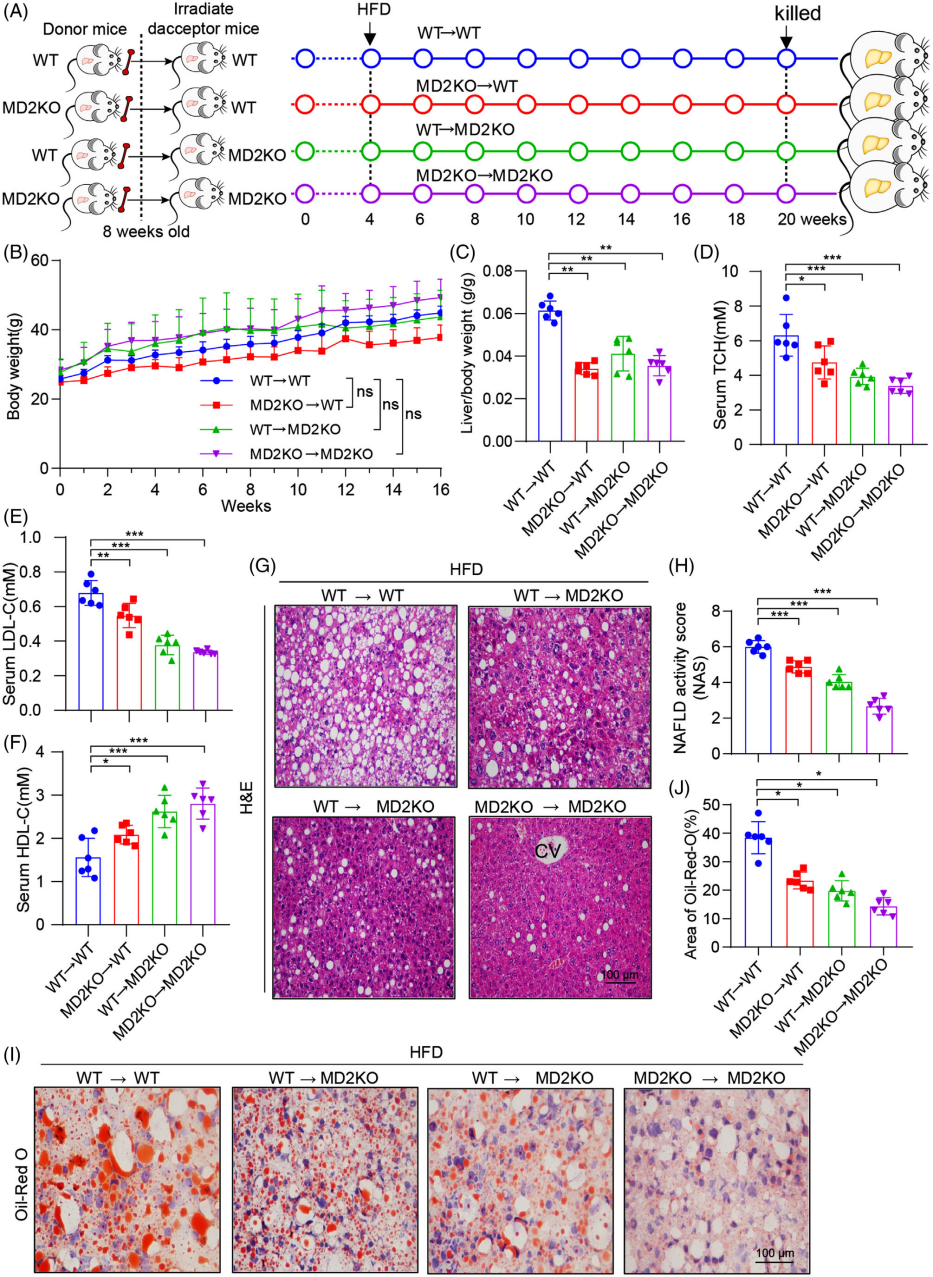
Myeloid and hepatocyte MD2 is required for lipid accumulation in mice fed a high‐fat diet (HFD). (A) The schematic diagram showing the grouping and steps of animal experiments. WT and MD2KO mice were irradiated and received bone marrow cells from either WT mice or MD2KO mice (WT→WT = marrow cells from WT mice transplanted in irradiated WT mice; MD2KO→WT = marrow cells from MD2KO mice transplanted in irradiated WT mice; WT→MD2KO = marrow cells from WT mice transplanted in irradiated MD2KO mice; MD2KO→MD2KO = marrow cells from MD2KO mice transplanted in irradiated MD2KO mice). All transplanted mice were fed an HFD for 16 weeks. (B) Body weight of mice recorded weekly. (C) Liver weight relative to BW (LW/BW) ratios at the time of harvest. (D–F) Serum levels of total cholesterol (TCH) (D), low‐density lipoprotein‐cholesterol (LDL‐C) (E) and high‐density lipoprotein‐cholesterol (HDL‐C) (F) in mice after marrow reconstitution and HFD feeding for 16 weeks. (G and H) Hematoxylin and eosin (H&E) staining of liver tissues (G) and non‐alcoholic fatty liver disease (NAFLD) activity score analysis (H). (I and J) Lipid accumulation in liver tissues as detected by oil red O staining (I) (scale bar = 100 μm). Quantification of lipid area is shown in panel (J). In panel (C–J), *n* = 6 per group; data are shown in mean ± SEM; ns, not significant; *p < .05, **p < .01 and ***p < .001

To understand the role of hepatocyte MD2 versus myeloid cell MD2, we performed bone marrow reconstitution studies. Specifically, we irradiated WT mice and MD2KO mice to deplete their marrows and reconstituted with either WT or MD2KO marrow cells. We confirmed the high efficiency of bone marrow depletion and reconstitution through examining the levels of MD2 in liver tissues and BMDM of mice from four groups (Figure S3). Following irradiation and bone marrow cell transplantation, mice in all four groups were fed with HFD for 16 weeks (Figure 4A). BW gain was not different among the various combinations (Figure 4B). However, LW/TL ratios showed that replenishing wildtype marrow cells in MD2KO mice or replenishing MD2KO marrow cells in wildtype mice (correcting MD2 in myeloid and hepatocytes, respectively) did not increase tissue weights (Figure 4C), suggestive of liver protection. Serum lipid profile confirmed that both hepatocyte and circulating myeloid cell MD2 deletion afforded protection against hyperlipoidemia (Figure 4D–F). In agreement, H&E‐stained sections showed that MD2KO marrow cell transplantation in WT mice or wildtype marrow transplantation in MD2KO mice reduces hepatic NAS and steatosis (Figure 4G,H). The same effect was seen in oil red O staining for lipid accumulation (Figure 4I,J). These studies show that both hepatocyte and macrophage MD2 is involved in the pathogenesis of NALFD, confirming that, besides macrophage MD2, the hepatocyte MD2 also contributes to NALFD.

### 3.3 MD2 regulates lipid metabolism in liver tissues of mice fed an HFD through AMPK/SREBP

**FIGURE 5:**
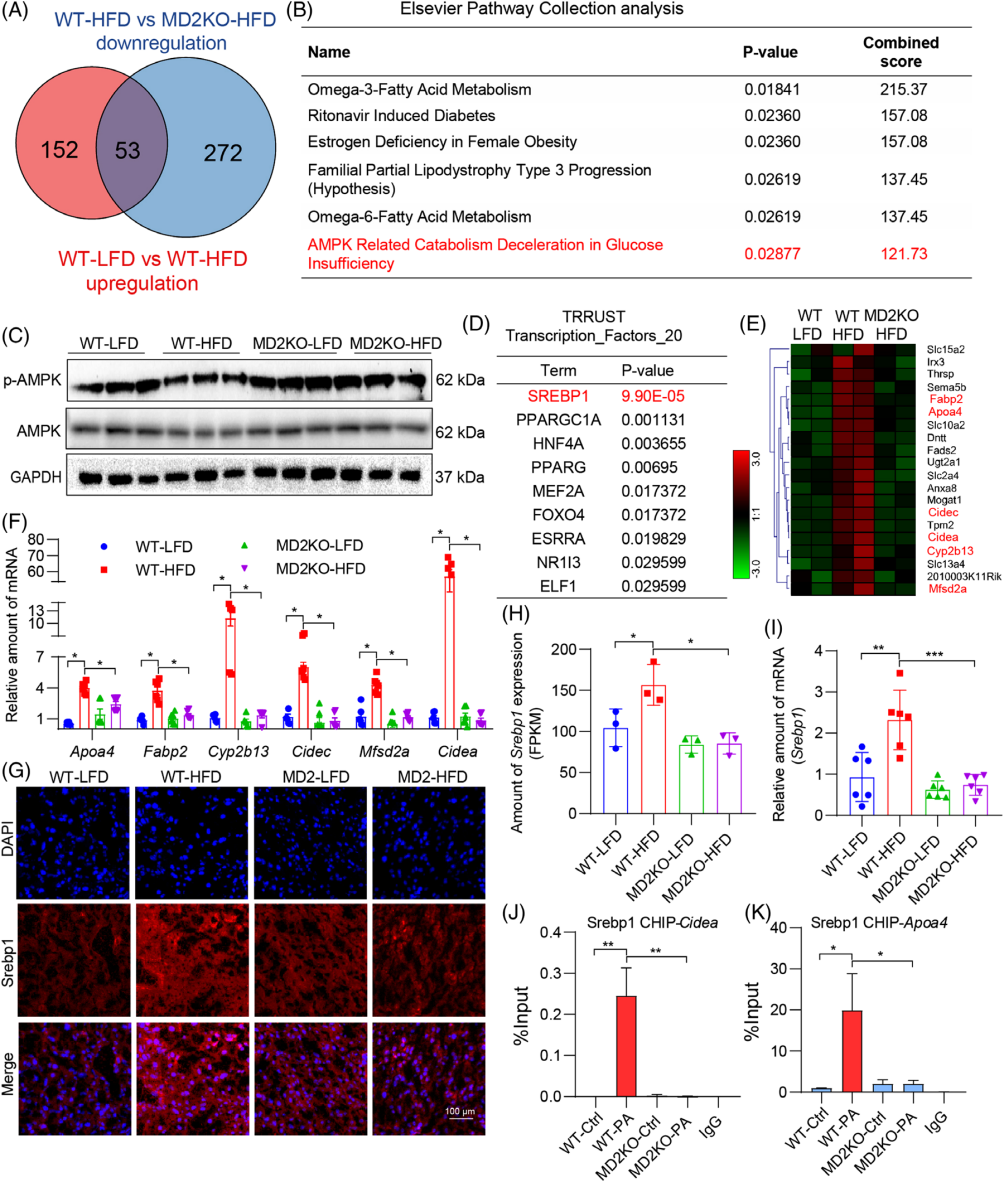
Identification of AMPK/sterol regulatory element binding protein 1 (SREBP1) downstream of MD2 in lipid accumulation. (A) Venn diagram of genes up‐regulated in wildtype mice fed a high‐fat diet (WT‐HFD) compared to WT fed a control diet (WT‐low‐fat rodent diet [LFD]) (red), and genes down‐regulated in MD2KO mice fed an HFD compared to WT fed HFD (blue). (B) Elsevier pathway collection analysis of genes showing increased levels in WT‐HFD but restored in MD2KO mice fed an HFD. (C) Levels of AMPK and phosphorylated (p‐) AMPK in mouse liver tissue lysates. GAPDH was used as loading control. (D) Transcription factor motifs enriched in the subset of HFD‐induced genes whose expression is restored in MD2KO mice (53 genes) using TRRUST transcription_factors. (E) Heat map showing relative expression of SREBP1 target genes from panel A. (F) Select SREBP1 target genes were assessed by real‐time qPCR. Levels were normalized to *Actb* (*n* = 6; mean ± SEM; *p < .05). (G) Representative immunofluorescence staining of liver tissues for SREBP1 (red). Tissues were counterstained with DAPI (blue) (scale bar = 100 μm). (H) Levels of Srebp1 in liver tissues as detected by RNA‐sequencing from panel A (*n* = 3; mean ± SEM; *p < .05). (J) Levels of Srebp1 in liver tissues as detected by real‐time qPCR. Data normalized to *Actb* (*n* = 6; mean ± SEM; **p < .01 and ***p < .001). (J and K) Chromatin IP‐qPCR analysis of SREBP1 target gene promoters showing *Cidea* (J) and *Apoa4* (K). Primary hepatocytes from WT and MD2KO mice were isolated and exposed to 200 μM palmitate (PA). IgG was used as negative control IP (*n* = 3; mean ± SEM; *p < .05 and **p < .01)

To understand how hepatocyte MD2 may participate in NAFLD, we performed RNA‐sequencing on liver tissues of the four mice groups in Figure 2. We examined genes up‐regulated in wildtype mice when fed an HFD, and compared these to genes down‐regulated/or not induced when MD2KO mice were fed an HFD (Figure S4A–D). Using this comparison, we identified 53 genes (Figure 5A). Pathway analysis revealed that these genes regulate lipid metabolism (Figure 5B, Figure S5). Of these pathways in Figure 5B, AMPK piqued our interest as AMPK is a main and well‐known regulating protein for lipid metabolism, and AMPK activation has been proposed to be therapeutically beneficial for NAFLD.^27^ We examined mouse liver tissue lysates from these four groups for AMPK phosphorylation as a proxy for protein activation. We noted reduced p‐AMPK levels in WT‐HFD mouse liver but increased p‐AMPK levels in MD2KO mice, both on LFD and HFD (Figure 5C, Figure S4E). This suggests that lack of MD2 prevents HFD‐induced AMPK suppression and subsequent lipid accumulation in liver tissues.

Analysis of transcription factors associated with the 53 candidate genes highlighted sterol regulatory element‐binding protein 1 (SREBP1), a well‐recognized downstream transcriptional factor regulating hepatic lipid metabolic genes in the AMPK pathway^28^ (Figure 5D). This transcription factor was top ranked in TRRUST analysis, likely due to several SREBP1 targets in the 53 gene list (Figure 5E). We validated six selected target genes, which have been shown to regulate hepatic lipid metabolism,^29^ by qPCR assay. The results showed that MD2KO mice do not show the same induction of these SREBP1 targets as WT mice when fed an HFD (Figure 5F). Furthermore, the target genes also showed reduced induction in liver tissues of mice with reconstituted marrow cells (Figure S6), in the similar changing trend with lipid profile. Staining of liver tissues showed robust SREBP1 immunoreactivity, while WT mice on HFD showed the most intense staining (Figure 5G). To provide quantitative assessment of *Srebp1* expression, we examined our RNA‐seq data and performed qPCR (Figure 5H,I). Both assays showed induction of *Srebp1* in wildtype mice on HFD but not MD2KO mice. It has been reported that AMPK activation could down‐regulate SREBP1 mRNA expression,^30^ suggesting that MD2KO inhibits *Srebp1* transcription possibly through AMPK activation. Next, we performed chromatin immunoprecipitation‐quantitative polymerase chain reaction (ChIP)‐qPCR to empirically show SREBP1 activity. For this study, the primary hepatocytes were isolated from wildtype and MD2KO mice (Figure S7A) and exposed to PA. This in vitro platform of PA challenge has been extensively used to model HFD in experimental models including NAFLD.^11^ Exposure of wildtype hepatocytes to PA increased *Md2* transcription (Supplementary Figure S7B), similar to the in vivo model of HFD. We further show that PA challenge of wildtype hepatocytes increases SREBP1 binding to promoter regions of *Cidea* and *Apoa4* (Figure 5J,K). This binding was not observed when we challenged MD2KO‐derived hepatocytes to PA, indicating that MD2 deficiency inhibited PA‐increased SREBP1 activity.

**FIGURE 6:**
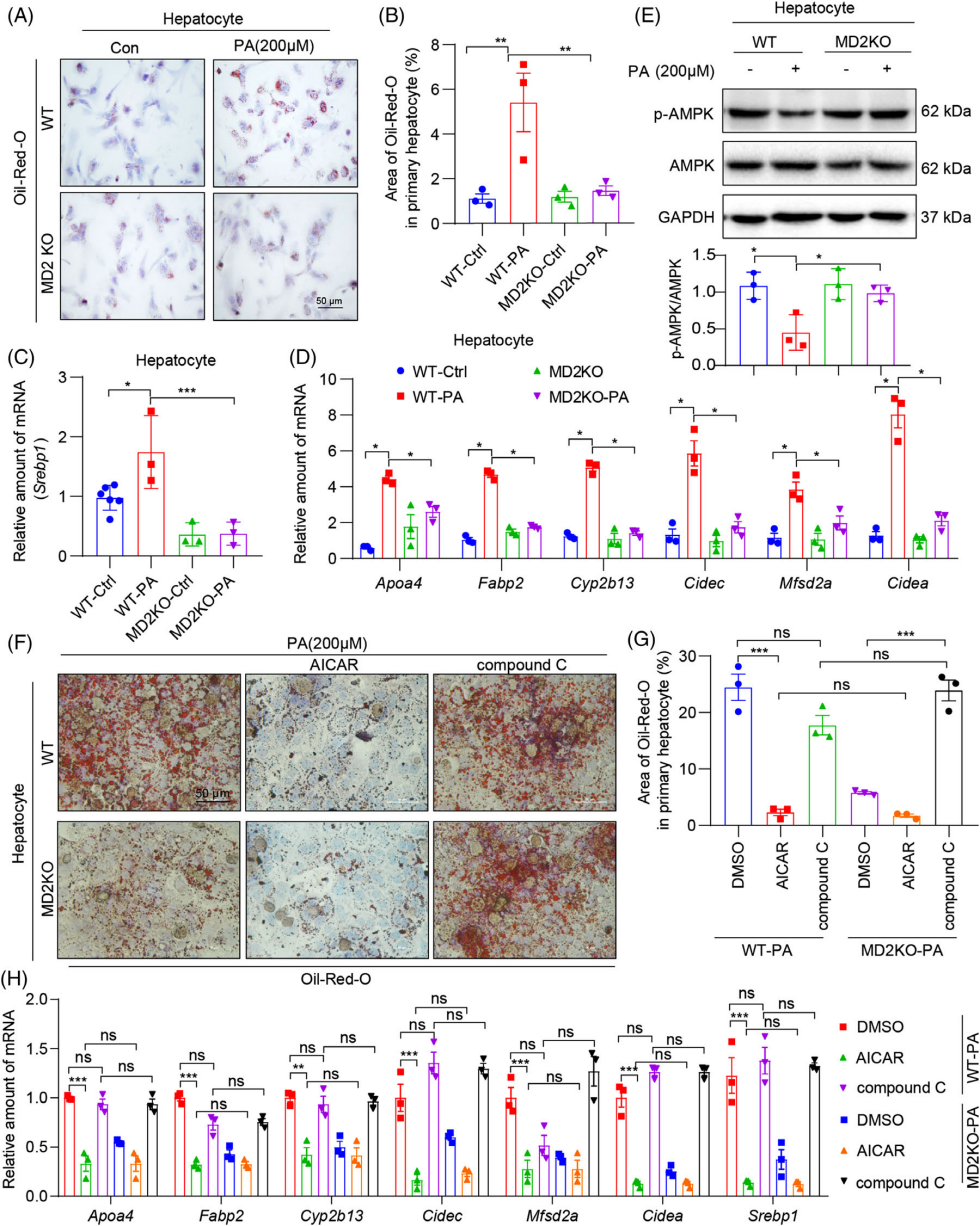
MD2 deficiency and AMPK activation reduced palmitate (PA)‐induced lipid accumulation in primary hepatocytes. Primary hepatocytes isolated from WT and MD2KO mice were exposed to 200 μM PA for 48 h. (A and B) Levels of intracellular lipids as detected by oil red O staining. Representative staining images are shown in A, and quantification is shown in B (scale bar = 50 μm). (C and D) mRNA levels of *Srebp1* (C) and sterol regulatory element binding protein 1 (SREBP1) target genes (C) in primary hepatocytes. Transcripts normalized to *Actb*. (E) Levels of p‐AMPK in hepatocytes as determined by immunoblotting. GAPDH was used as loading control. Densitometric quantification is shown in the lower panel. (F and G) Oil red O staining of primary hepatocytes. Primary hepatocytes from WT or MD2KO mice were pretreated with 10 μM 5‐aminoimidazole‐4‐carboxamide riboside (AICAR) or compound C for 1 h before exposure to 200 μM PA for 48 h. Panel (F) shows representative staining images, and panel G shows quantification of lipid area (scale bar = 50 μm). (H) Primary hepatocytes were treated as indicated in panel (F). The mRNA levels of *Apoa4, Fabp2, Cyp2b13, Mfsd2a, Cidec, Cidea* and *Srebp1* in hepatocytes were examined by qPCR assay. In all panels, *n* = 3 independent experiments; data are shown in mean ± SEM; *p < .05, **p < .01 and ***p < .001

To elucidate the role of MD2 in hepatocytes, we also carried out an RNA‐sequencing in PA‐challenged primary hepatocytes from WT or MD2KO mice. One hundred six genes appeared to be regulated by MD2 in PA context through these comparisons (Figure S8A–E). Pathway analysis on these 106 genes shows that fatty acid synthesis and adipogenesis pathways are regulated in MD2 deletion (Figure S8F,G). These results are consistent with the in vivo study and validate that MD2 regulates PA‐induced lipid metabolic dysfunction in hepatocytes. We next measured lipid accumulation in cultured hepatocytes in response to PA. Increased oil red O staining was observed in wildtype primary hepatocytes but not MD2KO‐derived cells (Figure 6A,B). Furthermore, AMPK phosphorylation was suppressed, and expression of *Srebp1* and SREBP1‐target genes was induced in WT hepatocytes exposed to PA (Figure 6D,E). As expected from our in vivo studies, primary hepatocytes from MD2KO mice did not show lipid accumulation, p‐AMPK suppression or induction of SREBP1 pathway. Since AMPK/SREBP1 pathway is highly related to mitochondria metabolism and function, we also examined the effects of MD2 knockout on oxidative phosphorylation in hepatocytes. As expected, PA significantly inhibits mitochondrial respiration in primary hepatocytes from WT mice, while MD2 knockout reversed PA‐induced mitochondrial dysfunction (Figure S9). We then activated AMPK using a well‐known compound AICAR and inhibited AMPK using a specific inhibitor compound C.^31^ We show that lipid uptake in wildtype hepatocytes is prevented by either AICAR or MD2KO after exposure to PA, and interestingly, AMPK inhibition by compound C reversed MD2KO‐inhibited lipid accumulation (Figure 6F,G). Even *Srebp1 and* SREBP1‐target gene induction was prevented by AICAR treatment of hepatocytes (Figure 6H), validating the AMPK/SREBP1 pathway. Compound C also reversed MD2KO‐inhibited SREBP1‐traget gene expression (Figure 6H). These studies functionally link MD2 to AMPK/SREBP1 in lipid accumulation in hepatocytes.

### 3.4 MD2 links HFD/PA to AMPK/SREBP1 through TBK1

**FIGURE 7:**
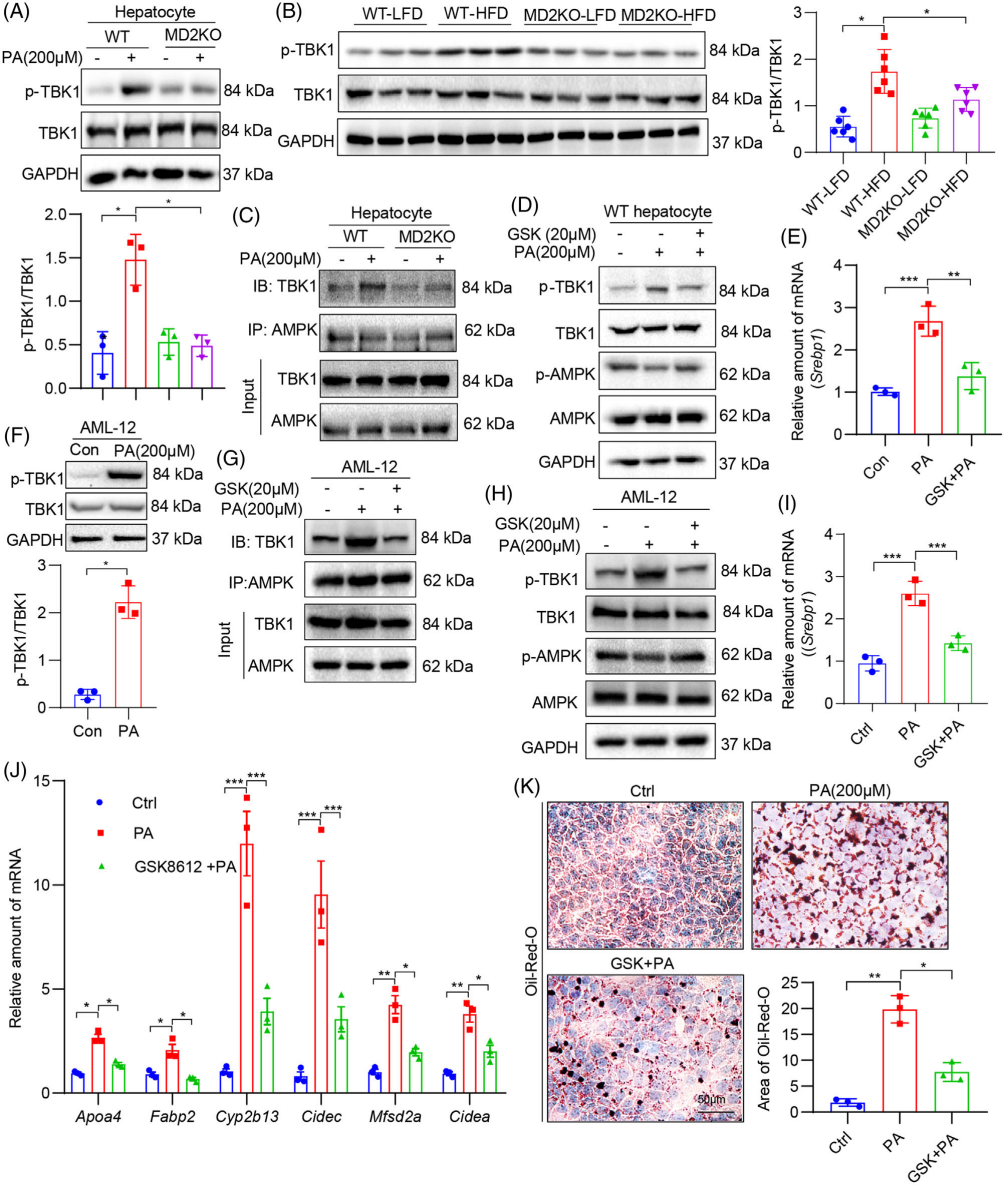
Lack of palmitate (PA) response in hepatocytes of MD2 knockout mice is through suppressed TBK1.(A) Primary hepatocytes isolated from WT mice and MD2KO mice were treated with 200 μM PA for 1 h. Levels of p‐TBK1 were determined by immunoblotting. GAPDH was used as loading control. Densitometric quantification is shown in lower panel. (B) Liver tissue lysates of WT and MD2KO mice fed a high‐fat diet (HFD) for 16 weeks were probed for p‐TBK1. GAPDH was used as loading control. Densitometric quantification is shown in right panel. (C) Immunoprecipitation of AMPK was performed in hepatocytes from WT and MD2KO mice after exposure to 200 μM PA for 1 h. Levels of TBK1 were detected by immunoblotting. (D) Primary hepatocytes from WT mice were pretreated with 20 μM GSK8612 (GSK) for 1 h before exposure to 200 μM PA for 1 h. Levels of p‐TBK1 and p‐AMPK were detected. GAPDH was used as loading control. (E) mRNA levels of Srebp1 in cells pretreated with 20 μM GSK for 1 h before exposure to 200 μM PA for 48 h. Data normalized to *Actb*. (F) AML‐12 cell line was exposed to 200 μM PA for 1 h. Levels of p‐TBK1 were detected. GAPDH was used as loading control. Densitometric quantification is shown on right. (G and H) AML‐12 cells were pre‐treated with GSK for 1 h and then challenged with PA for 1 h. TBK1‐AMPK complex was examined by immunoprecipitation (G). Whole cell lysates were probed for p‐TBK1 and p‐AMPK (H). (I and J) AML‐12 cells were pretreated with 20 μM GSK for 1 h before exposure to 200 μM PA for 48 h. mRNA levels of *Srebp1* (I) and sterol regulatory element binding protein 1 (SREBP1)‐target genes (J) were measured. Data normalized to *Actb*. (K) AML‐12 cells were treated as indicated in panel I. Lipid accumulation was detected by oil red O staining (scale bar = 50 μm). Representative images are shown in panel K. Quantification of lipid area is shown in lower panel. In all panels, *n* = 3 independent experiments; data are shown in mean ± SEM; *p < .05, **p < .01 and ***p < .001]

Our next objective was to determine how MD2 regulates AMPK, in the context of HFD in mice and PA exposure in vitro. We found that MD2‐regulated AMPK in NAFLD is independent to stress protein Sirtuins, since both RNA‐seq data and qPCR assay showed no change on Sirtuins gene expression (Supplementary Figure S10). A recent study showed that the noncanonical IKK family member TBK1 can directly inhibit AMPK to regulate energy homeostasis.^32^ TBK1 is a well‐established downstream mediator of TLR/MD2 pathway.^33^ Thus, TBK1 may represent such a mediator of AMPK regulation in HFD/PA models. Indeed, exposure of wildtype hepatocytes to PA increased p‐TBK1 levels (Figure 7A). As expected, hepatocytes from MD2KO mice did not show increased p‐TBK1. We also examined the transcripts of three TBK1‐downstream genes, *Ilb, Tnf* and *Dr5*. As shown in Figure S11A, MD2 deletion significantly reversed PA‐induced these gene expression. Similar results were obtained when we probed liver lysates from mice. HFD feeding of wildtype mice increased p‐TBK1, while the levels were significantly diminished in MD2KO mice (Figure 7B). Co‐immunoprecipitation assays showed increased TBK1‐AMPK association in wildtype hepatocytes exposed to PA (Figure 7C). The levels of TBK1 associating with AMPK were significantly lower in MD2KO hepatocytes.

We then inhibited TBK1 using a selective small‐molecule inhibitor GSK8612^34^ in PA‐challenged mouse primary hepatocytes. We noted significantly reduced p‐TBK1 and restored p‐AMPK by GSK8612 treatment (Figure 7D, Figure S11B). Downstream of AMPK restoration, levels of *Srebp1* transcripts were significantly reduced by GSK8612 (Figure 7E). Identical results were obtained with a mouse hepatocyte line, AML‐12 (Figure 7F–I, Figure S11C). We further examined SREBP1‐target genes (Figure 7J) and lipid accumulation (Figure 7K) in the cell line and showed that TBK1 inhibition by GSK8612 protected cells from PA‐induced SREBP1 activation and lipid accumulation.

### 3.5 Inflammatory cytokines from macrophages also contribute to AMPK/SREBP alteration in HFD/PA‐challenged hepatocytes

**FIGURE 8:**
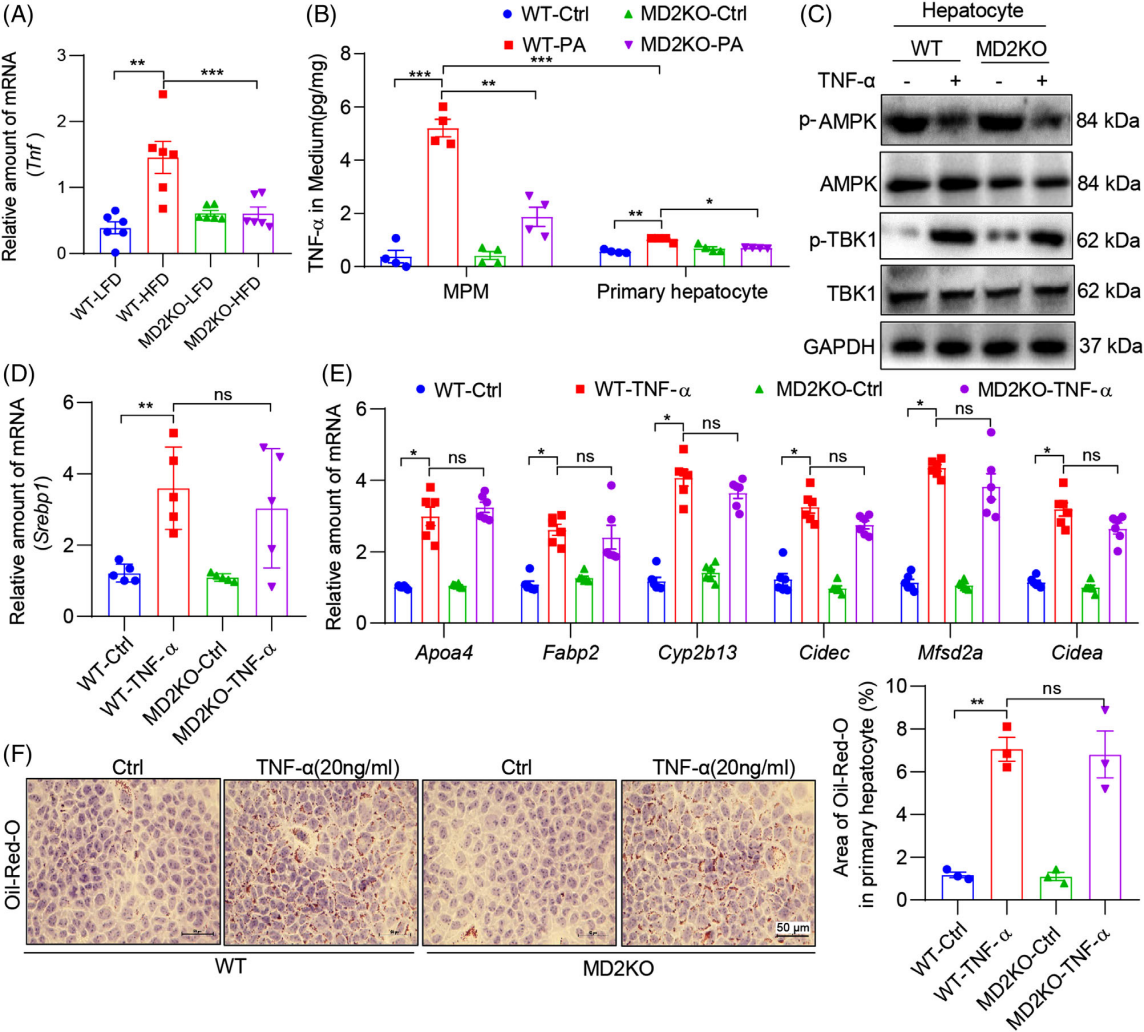
Tumour necrosis factor‐α (TNF‐α)‐α, induced by palmitate (PA) and MD2, activates TBK1 to suppress AMPK. (A) mRNA levels of *Tnfa* in liver tissues of WT and MD2KO mice fed a high‐fat diet (HFD) for 16 weeks (*n* = 6; mean ± SEM; **p < .01 and ***p < .001). (B) Mouse primary macrophages (MPM) and hepatocytes were isolated from WT and MD2KO mice. Cells were exposed to 200 μM PA for 48 h. Levels of TNF‐α protein in culture media was measured by ELISA (*n* = 4; mean ± SEM; **p < .01 and ***p < .001). (C) Primary hepatocytes were exposed to 20 ng/ml TNF‐α for 1 h. Levels of p‐AMPK and p‐TBK1 were detected by immunoblotting. GAPDH was used as loading control. (D) mRNA levels of Srebp1 in primary hepatocytes from WT and MD2KO mice that were exposed to 20 ng/ml TNF‐α for 48 h (*n* = 4; mean ± SEM; ns, not significant; **p < .01). (E) mRNA levels of sterol regulatory element binding protein 1 (SREBP1) target genes in cells treated as indicated in panel (D) (*n* = 6; mean ± SEM; ns, not significant; *p < .05). (F) Oil red O staining of cells exposed to TNF‐α (scale bar = 50 μm). Cells were treated as indicated in panel (D). Quantification of lipid area is shown in right panel (n = 3; mean ± SEM; ns, not significant; *p < .05)

Since our data in Figure 4 indicate MD2 in marrow‐derived myeloid cells also contributes partly to HFD‐induced lipid metabolism disturbance, we investigated the possibility that MD2‐mediated inflammatory factors in macrophages would contribute to TBK1‐AMPK/SREBP activation in our experimental systems. To study this, we first measured the expression of TNF‐α, a representative inflammatory cytokine, in liver tissues of wildtype and MD2KO mice. *Tnfa* expression was significantly increased in wildtype mice fed an HFD (Figure 8A). However, HFD‐induced Tnf over‐expression was normalized in liver tissues of MD2KO mice. We then measured TNF‐α protein in culture media of cultured primary hepatocytes or mouse primary macrophages (MPMs) following exposure to PA. While increased TNF‐α levels were seen in wildtype hepatocytes exposed to PA, the induction was not robust (Figure 8B). In addition, MD2KO‐derived hepatocytes failed to show significant change in TNF‐α level. Parallel analysis in MPMs harvested from the same mice induced that these cell types may be the primary source of increased TNF‐α in liver tissues (Figure 8B).

To examine the response of hepatocytes in the context of increased TNF‐α, we exposed the cells to TNF‐α and probed for AMPK and TBK1 activation. Our results show that TNF‐α induces p‐TBK1, suppresses p‐AMPK, increases the expression of *Ilb, Tnf, Dr5*, *Srebp1* and SREBP1‐target genes (Figure 8C–E, Figure S12A,B) and leads to increased lipid accumulation (Figure 8F). Interestingly, these changes were also seen in MD2KO‐derived hepatocytes, indicating the inductions by TNF‐α are independent of MD2. These results are in accordance with the previous report that TNF‐α induces TBK1 activation via TNF receptor 1, independent of MD2/TLR4,^35^ indicating that MD2‐mediated TNF‐α production will further regulate TBK1‐AMPK cascade via TNF‐α/TNFR1 pathway. In addition, MD2‐mediated other inflammatory cytokines in macrophages may also contribute to TBK1‐AMPK/SREBP activation and lipid accumulation in hepatocytes. Limiting the study in TNF‐α may be not very convincing. Therefore, we exposed mouse primary macrophages to PA and collected the conditional media to treat primary hepatocytes. As shown in Figure S12C–E, MD2 deficiency in macrophages reduced the conditional media‐induced lipid accumulation and over‐expression of SREBP1 target genes in hepatocytes. Collectively, these studies implicate macrophage‐derived inflammatory factors in contributing to hepatocyte lipid accumulation and injury, through TBK1‐AMPK/SREBP1 pathway.

## 4 DISCUSSION

In this study, we have shown for first time to our knowledge that MD2 directly regulates lipid metabolism in hepatocytes. Specifically, we show that the lack of MD2 in hepatocytes prevents HFD‐mediated suppression of AMPK1 and associated increase in downstream SREBP1. We further show that this protection is mediated, at least in part, through reduced TBK1 activation, a consequence of deficient *Md2*. Exposure of cultured hepatocytes to AMPK activator or TBK1 inhibitor mimics *Md2* deficiency. In addition, MD2‐mediated inflammatory factors in macrophages also contribute to hepatocyte lipid accumulation and injury. These studies have identified a novel mechanism by which MD2 plays a pathogenic role in NAFLD through TBK1‐AMPK/SREBP1 and lipid metabolism pathway (working model shown in the graphical abstract).

Studies show that a variety of factors are responsible for the development of NAFLD. Most of these discoveries have been made in mouse models upon HFD feeding.^36^ Earlier studies pointed to a critical role of MD2/TLR in mediating inflammatory response in NAFLD.^10^ This is not surprising because hepatocytes show transcripts of all TLRs.^37^, ^38^ Furthermore, engaging TLR2 or TLR4 through agonists activates nuclear factor‐κB in primary hepatocytes.^39^ Such responses cause production of the proinflammatory cascade and oxidative stress.^40^ These mechanisms are highly relevant to NAFLD and may cause progression of the disease to NASH. Deficiency of *Tlr4* has been shown to prevent liver damage against both steatosis and steatohepatitis.^13^, ^41^, ^42^ Saturated fatty acids in HFD may directly activate TLR through binding to MD2. In support of this mechanism, recently, we have reported that PA directly binds to MD2 to induce cellular injury.^12^ Therefore, a role of MD2 in linking HFD and PA to elaboration of inflammatory factors seems to be well established.

The novel finding of this study is linking MD2 to non‐inflammation‐related mechanism of liver injury. We know that the pathogenesis of NAFLD begins with triacylglycerol accumulation in the liver. Day and colleagues proposed that this represents the first hit in a two‐hit model.^43^, ^44^ The first hit is fat accumulation in the hepatocytes, which is followed by the second hit that causes inflammation, hepatocyte injury and fibrosis. In this continuum, depending on when analyses are performed, different factors may emerge as key contributors. Our analysis of published studies utilizing high‐throughput transcriptional changes in human NAFLD showed elevated *Md2* transcripts.^21^, ^22^, ^23^ Importantly, some of these studies were designed to probe for changes in early, non‐fibrotic NAFLD.^21^ This suggests that participation of MD2 in NAFLD pathology may start early. Our own RNA‐Seq studies showed key HFD‐induced metabolic pathways in wildtype mice are not induced in *Md2* deficient mice. This supports a potential role of MD2 during the potential first hit in the Day two‐hit model.^43^, ^44^ Recently, the ‘multiple‐hit’ hypothesis,^45^ which considers multiple causes acting together on genetically predisposed subjects to induce NAFLD, has been presented. It may provide a more accurate explanation for the pathogenesis of NAFLD. Future investigations should be carried out to assess the involvement of MD2 in other pathological processes of NAFLD.

Our studies have revealed that having intact MD2 allows HFD to suppress AMPK, a key regulator of energy metabolism in cells.^46^, ^47^ AMPK is regulated by various metabolic stresses. Classically, stresses that increase AMP/ATP ratios or other cellular states, such as excess glycogen, lipid and NAD/NADH redox potential^48^, ^49^, ^50^ regulate AMPK. A recent study, however, showed that the TBK1 may directly inhibit AMPK.^32^ Indeed, our studies confirmed this mechanism and demonstrated that lack of *Md2* prevented TBK1 activation and preservation of AMPK. Although this is the first documented evidence linking MD2 to AMPK, indirect evidence did exist in other systems. For example, *Tlr4*‐deficient non‐obese diabetic mice show increased p‐AMPK^51^ in heart tissues compared to wildtype non‐obese diabetic mice. Together, these studies provide a new function to MD2/TLR4 pathway.

Our studies showed robust expression of MD2 in both hepatocytes and F4/80 macrophage‐like cells in the liver. Hepatic macrophages consist of bone marrow‐derived monocytes migrating from the circulation into the liver and tissue‐resident KCs.^52^ In a healthy liver, the bone marrow‐derived macrophages only account for a small population of hepatic macrophages.^53^ However, these tissue‐resident and blood‐derived macrophages proportions change during liver injury. A prominent feature of any liver injury is that hepatic macrophage numbers increase.^54^ Using a well‐established model of marrow depletion and reconstitution, we show that both hepatocyte and marrow‐derived macrophage MD2 plays a role in lipid metabolism/accumulation and liver dysfunction. Mechanistically, macrophage activation by HFD increases inflammatory cytokine production, as we reported previously.^10^ In agreement with this notion, exposure of macrophages and hepatocytes to PA showed that macrophages were the primary source of proinflammatory factors. To investigate the consequence of an inflammatory environment, we exposed hepatocytes, with intact MD2 or without, to TNF‐α or multiple cytokines‐containing medium, and show suppressed AMPK, induced SREBP1 and lipid accumulation. These findings suggest that HFD/PA may activate MD2‐TBK1 directly in hepatocytes or through induction of macrophage‐derived inflammatory factors.

Although our studies have shown that macrophage‐derived inflammatory factors may suppress AMPK in hepatocytes, the converse situation may also be possible. In addition to a primarily metabolic role of AMPK, recent studies have shown additional functions of AMPK related to modulation of inflammatory responses in various cell types, including neutrophils^55^ and macrophages.^56^, ^57^ For example, AMPK suppresses the production of NF‐κB‐mediated cytokines in cells that have been stimulated to activate TLR4.^55, 57^ In addition, treatment of mice with the AMPK activator AICAR reduces the severity of lipopolysaccharide‐induced inflammatory lung injury.^55^, ^58^ Therefore, future studies dissecting the inflammatory and metabolic arm as well as the synergy between AMPK activation and MD2 inhibition would be valuable. AMPK also plays roles in myeloid cells, and our data still see significant effects of myeloid MD2 in NALFD. Thus, it would be valuable for future studies to examine the roles of MD2‐AMPK signaling pathway in myeloid cells.

In summary, we have reported a novel mechanism, by which MD2 contributes to NAFLD pathogenesis. In addition to the well‐documented role of MD2 in facilitating TLR4 activation and induction of inflammatory injury, we discovered that MD2 regulates AMPK activation through TBK1. Lack of MD2 in mice prevented HFD‐mediated inactivation of AMPK1 and increased SREBP1 and SREBP1 target gene expression. These changes manifested as reduced lipid accumulation and reduced liver injury. Importantly, we show that regulation of AMPK by MD2 in hepatocytes is mediated through TBK1. These studies link hepatocyte MD2 to lipid metabolic pathways in NALFD. Furthermore, our results suggest that MD2 may potentially be targeted for the development of NAFLD therapeutics.

## CONFLICT OF INTEREST

The authors declare that they have no conflict of interest

## EXPECTS DATA SHARING

The data that support the findings of this study are available from the corresponding author upon reasonable request.

## Supporting information

Supporting information.
Supplemental information includes two tables and 12 figures. All the other data are available from the authors on reasonable request.
