# Supplementary material for: MD2 deficiency prevents high‐fat diet‐induced AMPK suppression and lipid accumulation through regulating TBK1 in non‐alcoholic fatty liver disease
Source: Clin Transl Med. 2022 Mar 28;12(3):e777. doi: 10.1002/ctm2.777 (PMC8958353; doi:10.1002/ctm2.777)
Supplement: Supplementary file 1 — Supporting information. Supplemental information includes two tables and 12 figures. All the other data are available from the authors on reasonable request. [file CTM2-12-e777-s001.pdf]

Supporting information

MD2 deficiency prevents HFD-induced AMPK suppression and lipid accumulation through regulating TBK1 in nonalcoholic fatty liver disease

Wu Luo, Lin Ye, Xueting Hu, Meihong Wang, Minxiu Wang, Leiming Jin, Zhongxiang Xiao, Jianchang Qian, Wei Zuo, Lijiang Huang, Guang Liang

Supplemental information includes 8 figures and 2 tables.

Supplementary Table S1: The histological features and clinical information of NASH patients.

	Normal	NAFLD
All	6	6
Male Gender	3	2
Age (years)	45.3±6.2	55.3±2.2
BMI (kg/m2)	21.1±2.2	35.11±2.9
AST (U/L)	15.2±1.5	54.4±4.6
ALT (U/L)	25.4±2.1	80.1±5.1
Triglycerides (mg/dL)	120.5±13.5	220.5±19.8
Cholesterol (mg/dL)	125.4±11.7	240.5±25.1
HDL (mg/dL)	70.1±7.7	40.2±3.5
LDL (mg/dL)	69.5±7.5	135.5±12.9
Steatosis grade (1/2/3)	0	2/1/3
Fibrosis (0/1)	0	1/5
Lobular inflammation (0/1/2/3)	0	1/2/3

Supplementary Table S2: Primer sequences for real-time qPCR assay in this study.

Gene Name	Species	Application	Primer sequence (5'-3')	
Actb	Mouse	qPCR	Forward	CCGTGAAAAGATGACCCAGA
			Reversed	TACGACCAGAGGCATACAG
Md2	Mouse	qPCR	Forward	CGCTGCTTTCTCCCATATTGA
			Reversed	CCTCAGTCTTATGCAGGGTTCA
Srebp-1	Mouse	qPCR	Forward	TGACCCGGCTATTCCGTGA
			Reversed	CTGGGCTGAGCAATACAGTTC
Apoa4	Mouse	qPCR	Forward	CCAATGTGGTGTGGGATTACTT
			Reversed	AGTGACATCCGTCTTCTGAAAC
Fabp2	Mouse	qPCR	Forward	GTGGAAAGTAGACCGGAACGA
			Reversed	CCATCCTGTGTGATTGTCAGTT
Mfsd2a	Mouse	qPCR	Forward	AGAAGCAGCAACTGTCCATTT
			Reversed	CTCGGCCCACAAAAAGGATAAT
Cidea	Mouse	qPCR	Forward	TGACATTCATGGGATTGCAGAC
			Reversed	GGCCAGTTGTGATGACTAAGAC
Cidec	Mouse	qPCR	Forward	ATGGACTACGCCATGAAGTCT
			Reversed	CGGTGCTAACACGACAGGG
Cyp2b13	Mouse	qPCR	Forward	TTTTCTTCCAGTGTGTTACAGCC
			Reversed	AACGCAGGAACTGTTCATCTG
Tnf	Mouse	qPCR	Forward	TGATCCGCGACGTGGAA
			Reversed	ACCGCCTGGAGTTCTGGAA
Srit1	Mouse	qPCR	Forward	GCTGACGACTTCGACGACG

			Reversed	TCGGTCAACAGGAGGTTGTCT
Srit2	Mouse	qPCR	Forward	GCCTGGGTTCCCAAAAGGAG
			Reversed	GAGCGGAAGTCAGGGATACC
Srit4	Mouse	qPCR	Forward	GTGGAAGAATAAGAATGAGCGGA
			Reversed	GGCACAAATAACCCCGAGG
Ilb	Mouse	qPCR	Forward	TCGCAGCAGCACATCAACAAGAG
			Reversed	AGGTCCACGGGAAAGACACAGG
Dr5	Mouse	qPCR	Forward	CGGGCAGATCACTACACCC
			Reversed	TGTTACTGGAACAAAGACAGCC
Apoa4	Mouse	ChIP	Forward	AAAGTCACCCAGCCTGATGACA
			Reversed	GGCTACATAGAGAAACCCTGTC
Cidea	Mouse	ChIP	Forward	CTCTTGCAGGGCGTTCACT
			Reversed	TCTTCGCAAGCTACAAGGTCT

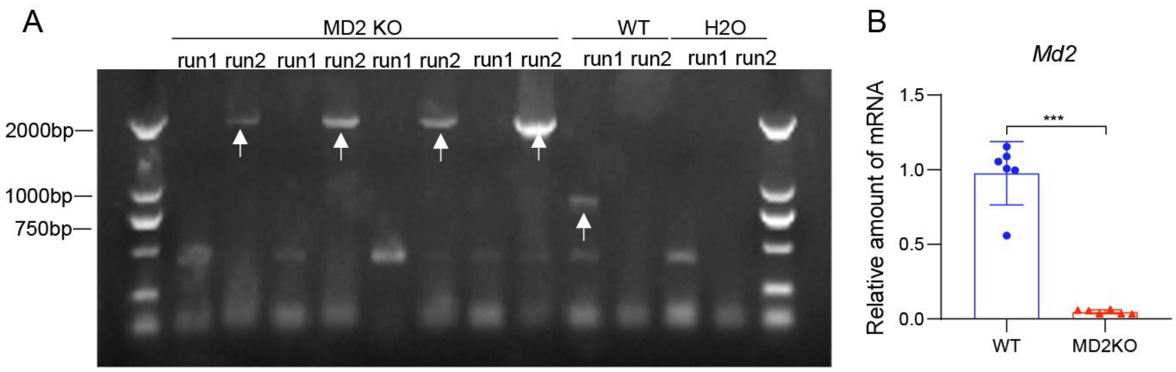

C

genotype	Species	Application	Primer sequence (5'-3')	
WT	Mouse	genomic DNA PCR	Forward	TGCCTTTAATTCCAGGAGGTAGAGG
			Reversed	ATGTTGCCATTTATTCTCTTTTCGA
MD2KO	Mouse	genomic DNA PCR	Forward	GGCCAGCTCATTCCTCCCACTC
			Reversed	GCCCAGGGAATTTTAGTAACTTGGGGC

Supplementary Figure S1: MD2 knockout confirmation in mice.

(A) Genotyping was performed using genomic DNA isolated from tail clip samples of 3-week-old mice. PCR products were separated using agarose gel electrophoresis to identify products of 800 bp ($MD2^{+/+}$), 1800 bp ($MD2^{-/-}$), and 800/1800 bp ($MD2^{+/-}$). (B) mRNA level of *Md2* in liver tissues of WT and MD2KO mice. Data was normalized to *Actb* [n = 6; Mean ± SEM; ***P < 0.001]. (C) The primers used to genotype analysis of MD2 knockout mice.

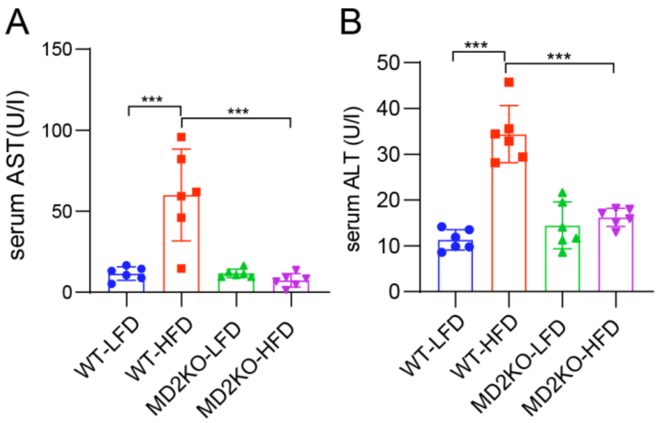

Supplementary Figure S2: Serum levels of liver enzymes in mice.

WT and MD2KO mice were fed with LFD or HFD for 16 weeks. Serum levels of aspartate aminotransferase (AST; A) and alanine aminotransferase (ALT; B) were measured using commercial kits [n = 6; Mean ± SEM; ***P < 0.001].

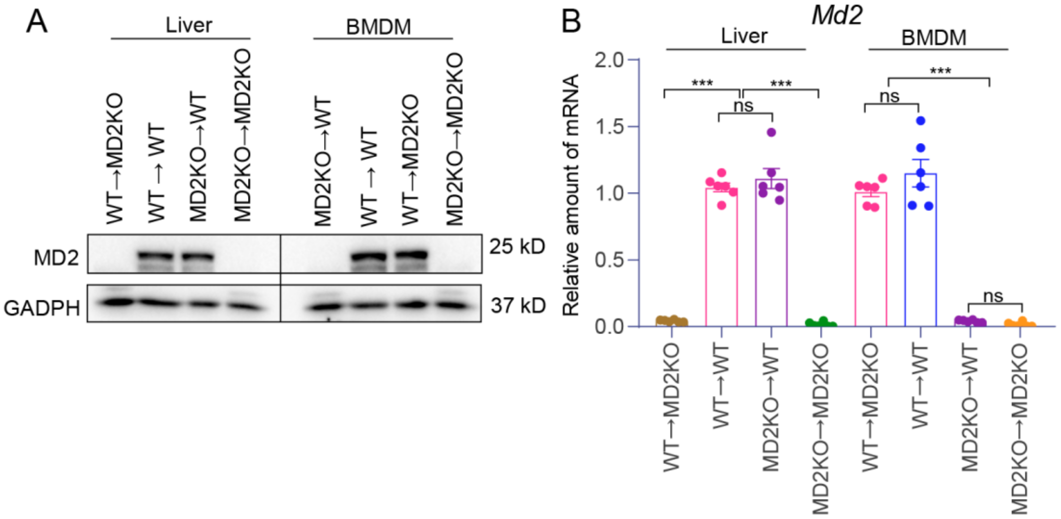

Supplementary Figure S3: MD2 knockout confirmation in bone marrow transplanted mice. WT and MD2KO mice were irradiated and received bone marrow cells from either WT mice or MD2KO mice [WT→WT = marrow cells from WT mice transplanted in irradiated WT mice; MD2KO→WT = marrow cells from MD2KO mice transplanted in irradiated WT mice; WT→MD2KO = marrow cells from WT mice transplanted in irradiated MD2KO mice; MD2KO→MD2KO = marrow cells from MD2KO mice transplanted in irradiated MD2KO mice]. All transplanted mice were fed an HFD for 16 weeks. The liver tissue and bone marrow-derived macrophages (BMDM) were collected from four groups when mice were killed. The protein (A) and mRNA (B) levels of MD2 in liver and BMDM of mice from these four groups were examined by Western blot and qPCR assay, respectively. [n = 6; Mean ± SEM; ns = not significant, ***p<0.001]

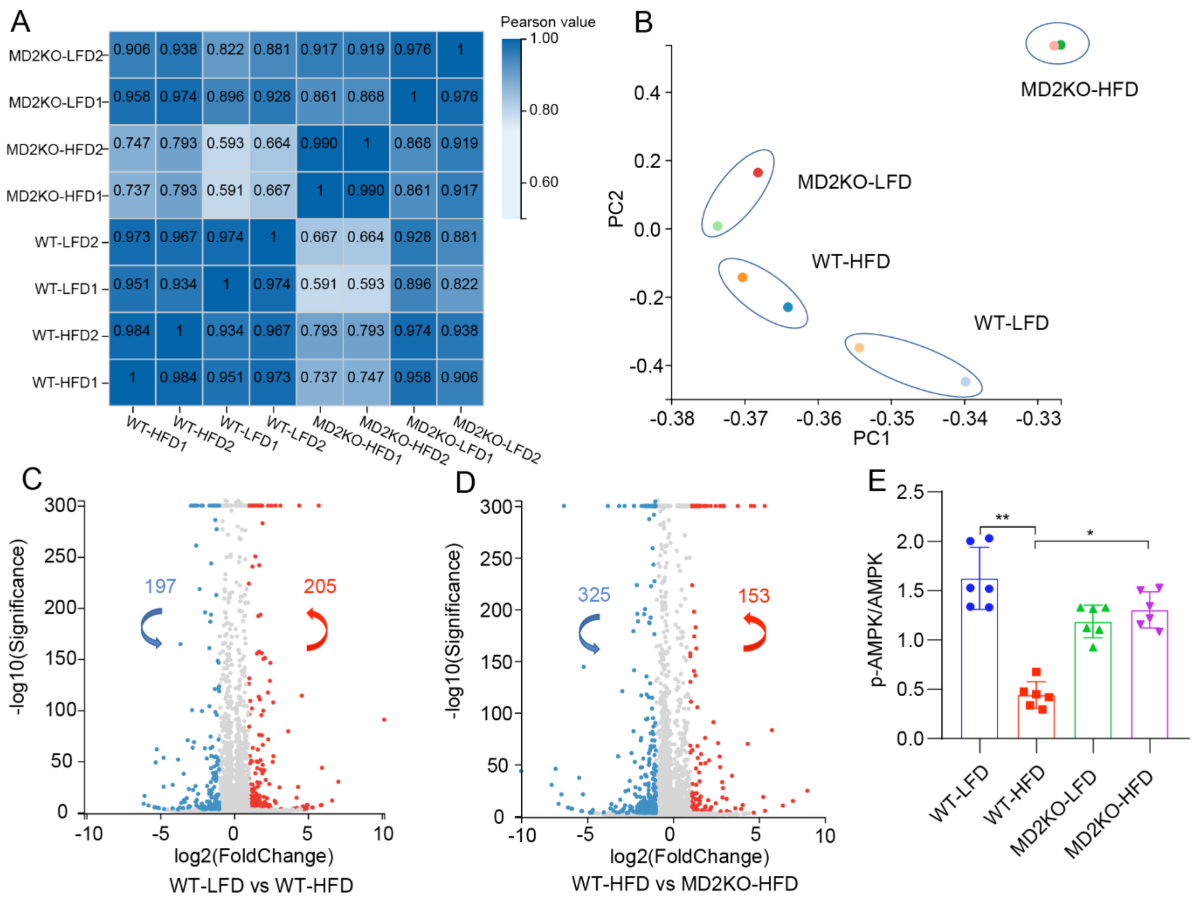

Supplementary Figure S4: Detection of MD2 regulates lipid metabolic pathways in HFD fed mice.

WT and MD2KO mice were fed with LFD or HFD for 16 weeks. Liver tissues were harvested for RNA-sequencing. (A) Pearson's correlation heatmap of all samples. (B) PCA analysis of RNA-sequencing data. (C, D) Volcano plot analysis of transcriptional changes in WT-LFD compared to WT-HFD (C), and WT-HFD compared to MD2KO HFD (D). The fold change threshold was set as 2 and P-value ≤ 0.01. Red dots indicate upregulated genes, blue dots indicate downregulated genes, and gray dots indicate non-differentially expressed genes. (E) Levels of p-AMPK proteins in liver tissues of mice. Densitometric quantification for blots in Figure 5C [n = 6; Mean ± SEM; *p<0.05, **p<0.01].

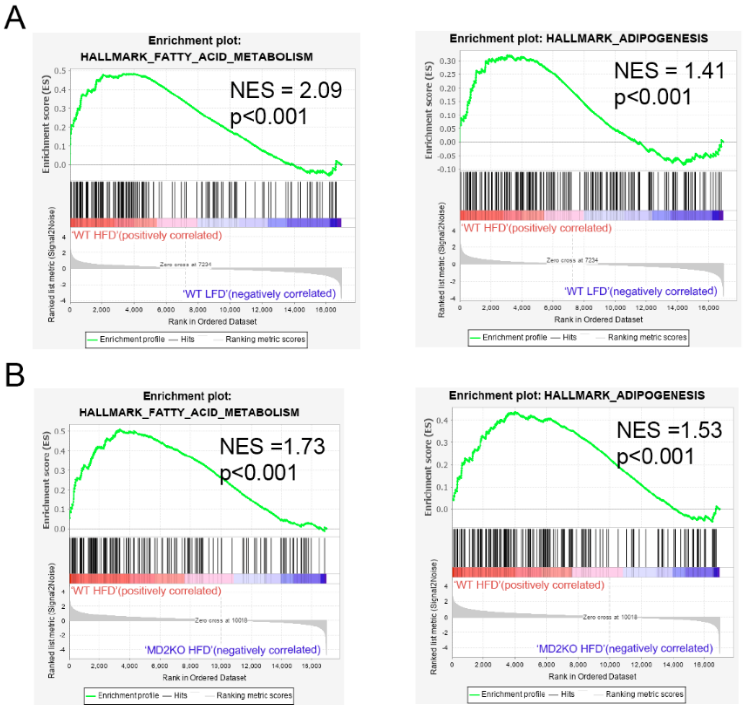

Supplementary Figure S5: GSEA enrichment analyses in liver tissues of wildtype and MD2KO mice.

WT and MD2KO mice were fed with LFD or HFD for 16 weeks. Liver tissues were harvested for RNA-sequencing. (A) GSEA showing significantly enriched signaling pathways or biological processes in the gene set upregulated in WT-HFD compared to WT-LFD. (B) GSEA showing significantly enriched signaling pathways or biological processes in the gene set upregulated in WT-HFD compared to MD2KO HFD.

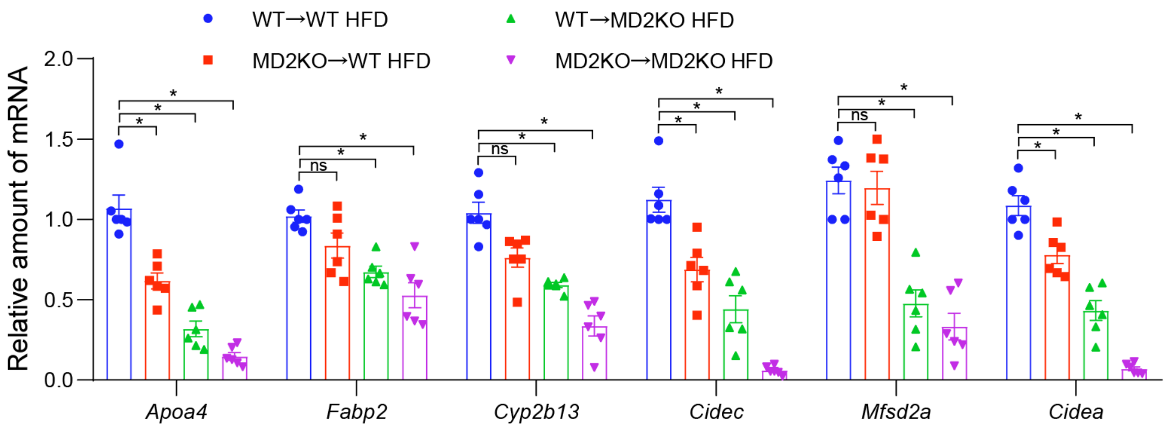

Supplementary Figure S6: mRNA levels of SREBP1 target genes in liver tissues of mice reconstituted with WT or MD2KO marrow and fed a HFD.

WT and MD2KO mice were irradiated and received bone marrow cells from either WT mice or MD2KO mice [WT→WT = marrow cells from WT mice transplanted in irradiated WT mice; MD2KO→WT = marrow cells from MD2KO mice transplanted in irradiated WT mice; WT→MD2KO = marrow cells from WT mice transplanted in irradiated MD2KO mice; MD2KO→MD2KO = marrow cells from MD2KO mice transplanted in irradiated MD2KO mice]. All mice were fed a HFD for 16 weeks. mRNA levels of SREBP1 target genes in liver tissues were measured by real-time qPCR assay. Data was normalized to *Actb* [n = 6; Mean ± SEM; *p<0.05].

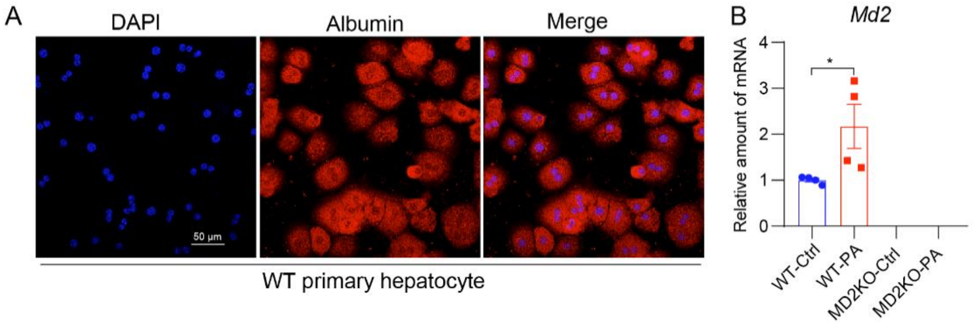

Supplementary Figure S7: Confirmation of primary hepatocyte isolation.

(A) Primary hepatocytes were isolated from liver tissues of mice. Albumin was detected by immunofluorescence staining (red). Cells were counterstained with DAPI (blue) [scale bar = 50 μm]. (B) Primary hepatocytes from WT and MD2KO mice were exposed to 200 μM PA for 48 h. mRNA levels of *Md2* were detected by qPCR. Data was normalized to *Actb* [n = 3; Mean ± SEM; *p<0.05].

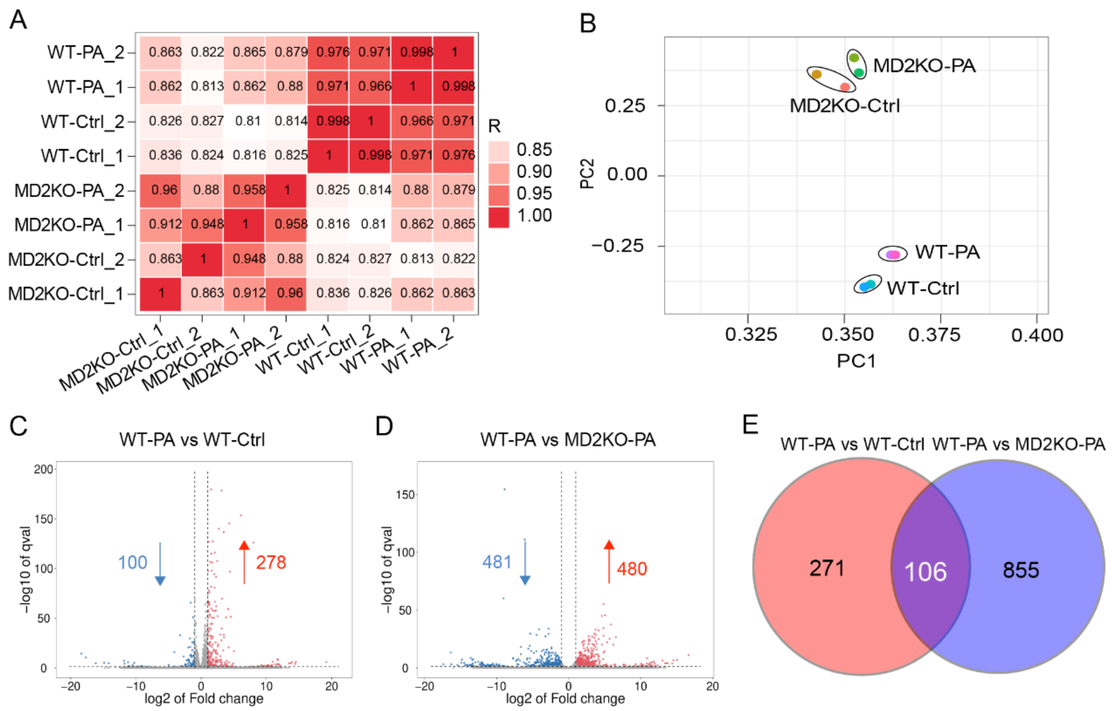

F MSigDB Hallmark 2020 pathway analysis

Name	P-value	Combined score
Bile Acid Metabolism	0.005189	18.60
Adipogenesis	0.03536	7.37
Xenobiotic Metabolism	0.03536	7.37
heme Metabolism	0.03536	7.37
Oxidative Phosphorylation	0.08332	4.76

G WikiPathway 2021 Human pathway analysis

Name	P-value	Combined score
Mitochondrial fatty acid synthesis pathway WP4317	0.003377	199.57
Methionine metabolism leading to sulfur amino acids and related disorders WP4292	0.0009647	137.31
NAD Biosynthesis II (from tryptophan) WP2485	0.009110	82.37
Methylation Pathways WP704	0.01157	67.01
Mitochondrial Gene Expression WP391	0.005069	52.23

Supplementary Figure S8: Detection of MD2 regulates metabolic pathways in mouse primary hepatocytes.

Primary hepatocytes from WT or MD2KO mice were exposed to 200 µM PA for 48 h. Cells were harvested for RNA-sequencing. (A) Pearson‘s correlation heatmap of all samples. (B) PCA analysis of RNA-sequencing data for all samples. (C, D) Volcano plot analysis of transcriptional changes in WT-Ctrl compared to WT-PA (C), and WT-PA compared to MD2KO-PA (D). The fold change threshold was set as 2 and P-value ≤ 0.05. Red dots indicate upregulated genes, blue dots indicate downregulated genes, and gray dots indicate non-differentially expressed genes. (E) Venn diagram of different genes in WT-Ctrl compared to WT-PA (red), and MD2KO-PA compared to WT-PA (blue). (F, G) The overlapped 106 genes in plane E are analyzed using MSigDB Hallmark 2020 pathway software and WikiPathway 2021 Human pathway analysis software, respectively, which shows that several metabolic pathways are involved.

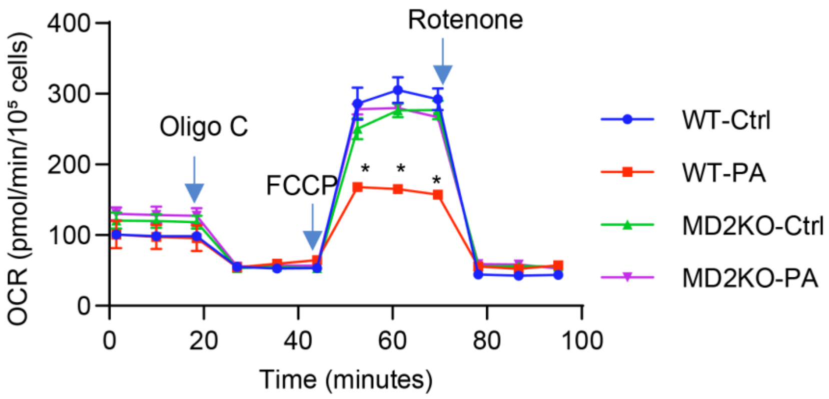

Supplementary Figure S9. MD2 knockout reversed PA-induced mitochondrial dysfunction. Primary hepatocytes isolated from WT mice or MD2KO mice were cultured with PA for 48 h, and OCR was measured in real time using the Seahorse XF96 Extracellular Flux Analyzer after basal OCR was measured at three time points, followed by sequential injection of oligomycin (1 μM), FCCP (0.5 μM), and rotenone (1 μM). The overall OCR curves were plotted as the mean OCR ± SD of three replicates.

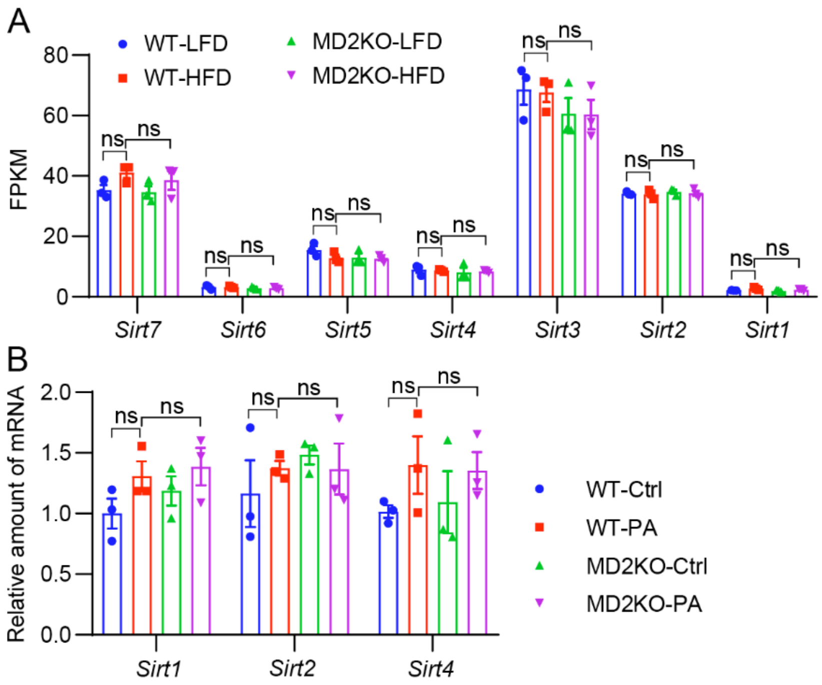

Supplementary Figure S10. MD2KO did not change the profile of Sirtuins gene expression. (A) Levels of Sirtuin (Sirt) 1~7 mRNA in liver tissues as detected by RNA-sequencing from Figure 5A [n = 3; mean ± SEM; *p<0.05]. (B) Primary hepatocytes from WT or MD2KO mice were exposed to 200 µM PA for 48 h. The mRNA levels of Sirt1, 2, and 4 were measured by qPCR, Data normalized to *Actb*. [n = 3; mean ± SEM; *p<0.05].

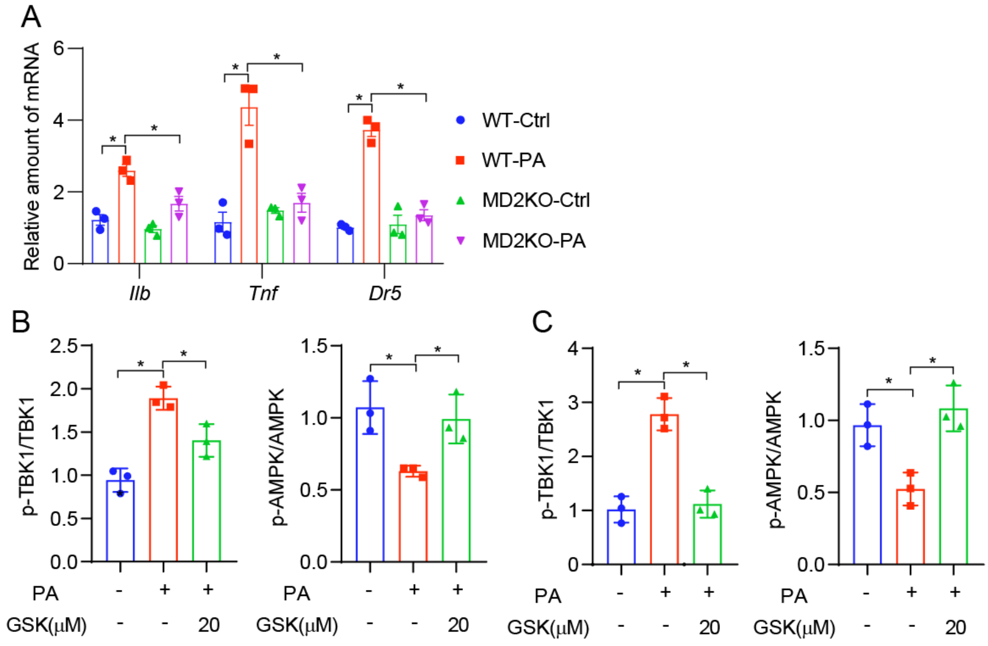

Supplementary Figure S11: TBK1 and AMPK signaling in liver tissues of mice fed a HFD and in hepatocytes exposed to PA.

(A) Primary hepatocytes isolated from WT and MD2KO mice were exposed to 200 μM PA for 12 h. The mRNA levels of *Ilb, Tnf,* and *Dr5* in primary hepatocytes were detected by qPCR. Data were normalized to *Actb*. (B) Densitometric quantification for blots in Figure 7D. (C) Densitometric quantification for blots in Figure 7H *p<0.05. [n = 3; Mean ± SEM; ns = not significant; *p<0.05].

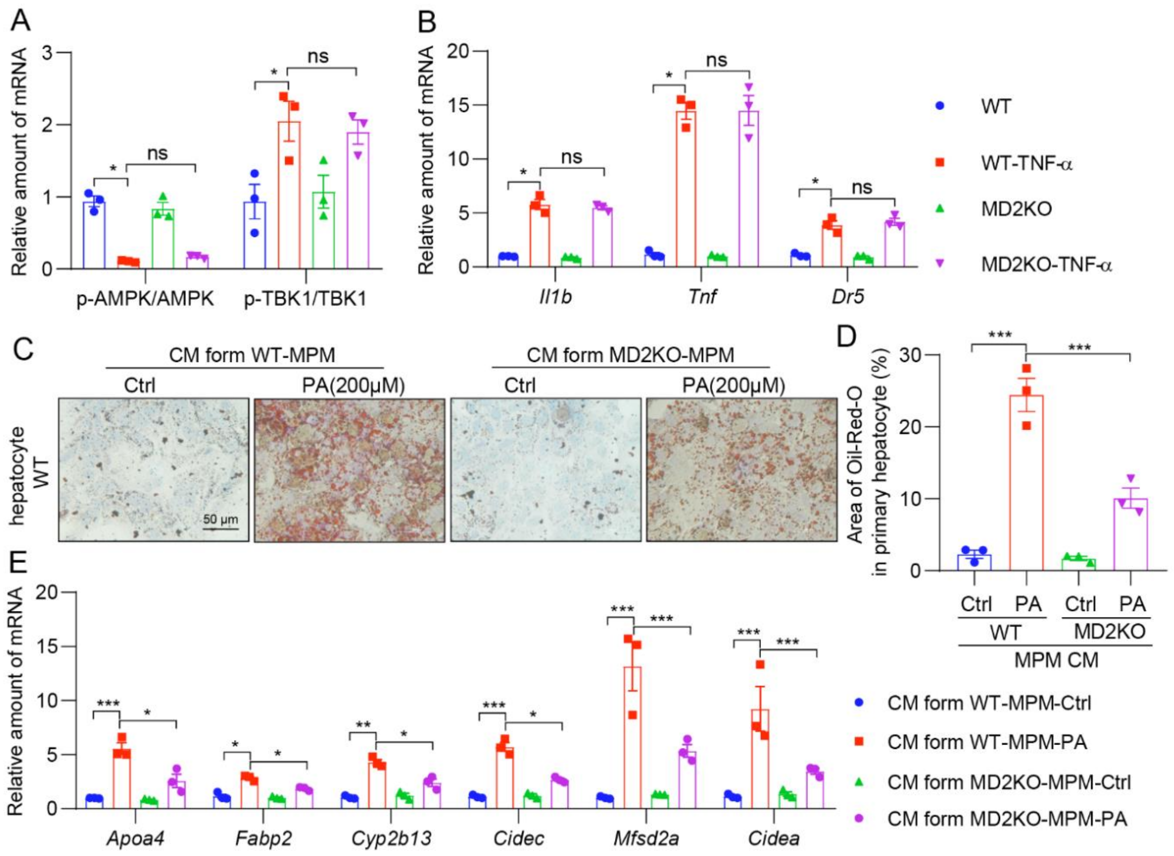

Supplementary Figure S12: MD2 deficiency in macrophages reduced the conditional media-induced lipid accumulation in hepatocytes. (A) Densitometric quantification for blots in Figure 8C. (B) Primary hepatocytes isolated from WT and MD2KO mice were exposed to 20 ng/mL TNF-α for 6 h. The mRNA levels of *Ilb, Tnf,* and *Dr5* in primary hepatocytes were detected by qPCR. Data were normalized to *Actb*. (C) Conditional media were collected from cultured WT or MD2KO mouse peritoneal macrophages treated with or without 200 μM PA for 48 h. Primary hepatocytes from WT mice were treated with the conditional medium (with fresh complete medium, 1:1) for 48 h. (D) The amounts of lipid droplets in cells were deleted by Oil Red O staining. The representative staining images and quantifications of lipid area were shown [scale bar = 50 μm]. (E) mRNA levels of *Apoa4, Fabp2, Cyp2b13, Mfsd2a, Cidec, Cidea* in primary hepatocytes exposed to conditional mediums were examined by qPCR assay. [n = 3; Mean ± SEM; ns = not significant; *p<0.05].
